# Genetic landscape of *Borrelia burgdorferi* sensu stricto in Canada: a study of genetic diversity

**DOI:** 10.1038/s41598-025-24758-2

**Published:** 2025-11-20

**Authors:** Samir Mechai, Edward J. Feil, Gabriele Margos, Nick H. Ogden

**Affiliations:** 1 Science and Policy Integration Branch, Applied Public Health Sciences Directorate, Public Health Agency of Canada, Saint-Hyacinthe, Québec J2S 2M2 Canada; 2 The Milner Centre for Evolution, Department of Life Sciences, University of Bath, Bath, UK; 3 National Reference Centre for Borrelia, Bavarian Health and Food Safety Authority, Oberschleissheim, Germany

**Keywords:** Phylogeny, Clonality, Modularity, Core genome, Accessory genome, Computational biology and bioinformatics, Diseases, Evolution, Genetics, Microbiology

## Abstract

*Borrelia burgdorferi* sensu stricto (s.s.), the causative agent of Lyme disease in North America, exhibits considerable genetic diversity. In order to gauge rates of recombination and the degree of geographically structuring within the population we carried out a comprehensive whole-genome comparison of *B. burgdorferi* s.s. strains (*n* = 64) across three Canadian regions - Nova Scotia (NS), Ontario (ON), and Manitoba (MB). Using a multi-marker approach (MLST, *ospC*, RSP, RST, IGS), we identified 12 genetically coherent groups that were stable across both core and accessory genome phylogenies. Our analyses reveal a clear geographic gradient of clonality, with NS harboring highly clonal and modular populations (clonal ratio = 4, modularity Q = 0.68), while ON/MB strains exhibited more recombination, shared markers, and genetic connectivity. Genes like *ospC* showed high recombination rates (R/θ = 4.25), whereas others (*ospA*, P45-13) evolved primarily via mutation (R/θ < 0.10), illustrating distinct selective pressures in host versus vector environments. Despite these differences, lineages remained phylogenetically robust across markers. These findings highlight how evolutionary processes shape the structure and diversity of *B. burgdorferi* s.s. populations in Canada and provide insights into its geographic spread and population ecology.

**Supplementary Information:**

The online version contains supplementary material available at 10.1038/s41598-025-24758-2.

## Introduction

*Borrelia burgdorferi* sensu stricto is the genospecies of the *B. burgdorferi* sensu lato complex with a wide geographic distribution in the northern hemisphere, including North America, and Europe^1^. The enzootic cycle typically involves small mammals and birds as competent reservoir hosts^2^. Humans are incidental hosts and do not contribute to onward transmission^3,4^. The geographic range of *I. scapularis* and *B. burgdorferi* s.s. has expanded northward in recent decades, especially into eastern and central Canada, raising public health concerns. *Borrelia burgdorferi* s.s. is the main bacterium that causes Lyme disease in North America, where it has a complex clonal structure as revealed using molecular methods such as multilocus sequence typing (MLST)^5,6^ and 16–23 S ribosomal RNA intergenic spacer (*rrs*-*rrlA*: IGS)^7^. Plasmid markers include the outer surface protein A (*ospA*) gene located on the lp54 plasmid^8^ and the outer surface protein C (*ospC*) gene of the cp26 plasmid^9^. These methods have uncovered a high level of diversity. Multilocus sequence typing (MLST) has resolved 180 sequence types (STs) in the USA and Canada to date^5^ (https://pubmlst.org/), while sequencing of the plasmid-located ospC gene has identified 47 types and subtypes of *B. burgdorferi* s.s. in North America^9–12^. Whilst these methods are not always consistent^5,13,14^, these data have shed light on key phenotypes such as disease severity in humans, reservoir host associations, and local adaptations. *ospC* alleles A, B, I, and K and RST1 were associated with disseminated Lyme disease, while *ospC* alleles J, T, U and RST3 are associated with more localized clinical symptoms of Lyme disease^15–17^. RST1, ST1, and *ospC* allele A have been found to be associated with white-footed mice, and RST2, and *ospC* G with eastern chipmunks, suggesting possible onset of specialization for these host species and evidence of radiative adaptation of *B. burgdorferi* s.s. in North America^18^. Variation between geographic regions has also been identified e.g., in the USA, ST1 is found exclusively in the Northeast, ST2 and ST5 in California, and ST55 and ospC allele A in the Upper Midwest^5,19^.

In North America, Lyme disease caused by *B. burgdorferi* s.s. has the highest incidence in the Northeastern and upper Midwestern regions of the United States. However, the phylogeographic patterns of this species reflect complex forces over millenia, and remain dynamic in the present day^20^. It is thought that anthropogenic changes to land use from woodland to agricultural land throughout the post-Columbian period, forced human-biting northern clades of *Ixodes scapularis* ticks^21,22^ and their key reproduction hosts (white-tailed deer), bird host, and key rodent reservoir hosts for *B. burgdorferi* s.s., into limited refugia in the upper Midwest and Northeast of the US producing a bottleneck for *B. burgdorferi* s.s. populations^23^. However, during the 20th century, industrialisation resulted in migration of human populations from agricultural communities to urban centres, and over time agricultural land has reverted to woodland and deer, rodent, tick and *B. burgdorferi* s.s. populations began to expand. By the late 1970’s Lyme disease was a significant public health issue in the northern US^24^. As well as land use change in the US, climate warming has rendered parts of Southern Canada climatically suitable for *I. scapularis* populations^25–27^.

This dynamic nature of *B. burgdorferi* s.s. populations over the last century has resulted in complex and varied population structures. Margos et al. (2012) identified that in the USA, *B. burgdorferi* s.s. populations are geographically structured into three sub-populations (northeastern, midwestern and western), with different clonal lineages being found in different regions. MLST data point to relatively recent introductions into Canada from multiple refugial southern populations in the USA^28,29^. In addition to geographic changes, the expansion of *B. burgdorferi* s.s. populations has been expected to undergo adaptive radiation, accompanied by multi-niche polymorphism^30^. *Borrelia burgdorferi* s.s. is a generalist capable of using a wide range of vertebrate species as reservoir host, but there is evidence of emerging host-genotype associations in North America^18^. Possible public health consequences of broadening diversity of *B. burgdorferi* s.s. include strain-specific manifestations of Lyme disease in affected people or impacts on sensitivity of serological diagnostic tests^31^.

The question is whether or not different genotyping methods can distinguish between these important phenotypes^32^. This is more likely the case with markers associated with the surface of the bacterium and which are carried on plasmids, such as OspC than with non-coding IGS sequences or other chromosomal markers. Here we explore the degree of consistency between plasmid and chromosomal markers.

An important related question concerns the extent of clonality within *B. burgdorferi* s.s. populations and how this influences their genetic structure and evolutionary dynamics. In the *B. burgdorferi* s.l. species complex, inter-specific recombination events are relatively uncommon^33^ compared to, for example, Enterobacteriaceae, possibly due to slow growth rates in hostile host and vector environments^33,34^. Nevertheless, recombination does occur, even on the chromosome^35^, although most recombination events are between closely related strains that are more likely to be adapted to the same host^31^.

In this study, we investigate these questions by analyzing the genome sequences of 64 *B. burgdorferi* s.s. strains collected in a previous study by Tyler and colleagues (2018) from three regions in Canada (Manitoba, Ontario, and Nova Scotia), representing a broad spectrum of genetic diversity and ecological contexts. Therefore, this is primarily a population genomics study investigating the phylogeography and population structure of *B. burgdorferi* in Canada, informed by genomic markers from both core and accessory genomes, with consideration of underlying evolutionary processes (recombination vs. mutation).

## Materials and methods

### Samples used and genome Preparation

Sixty-four *Borrelia burgdorferi* s.s. strains were isolated from host-seeking *Ixodes scapularis* ticks and sequenced by Tyler et al. (2018). Ticks were collected in 2016 by drag sampling in 10 locations in three Lyme-endemic regions of Canada: Buffalo Point and Roseau River in Manitoba; Big Grassy, Big Island, Birch Island, Manitou Rapids in northwestern Ontario; and Bedford, Lunenburg, Pictou, Shelburne in Nova Scotia. DNA libraries were prepared using TruSeq sample preparation kits (Illumina, San Diego, CA) and sequenced with 300 bp paired-end reads on the Illumina MiSeq platform. Genome assembly was performed using SPAdes v3.9 with contigs ≥ 1,000 bp. The complete genome sequences have been deposited in GenBank (BioProject accession number PRJNA416494).

### Gene panel selection

From the assembled genomes, we extracted genes representing both plasmid-encoded and chromosomal diversity:

**Plasmid-encoded genes**: Eleven surface-exposed protein-encoding genes important for Lyme disease diagnostics and pathogenicity: the C6 peptide (IR6 region of the *VlsE1* gene, lp28-1 plasmid in B31), *dbpA*, *dbpB*, fibronectin-binding protein P35, *oms28*, *ospA*, *ospB*, *ospC*, *ospD*, P37, and P45-13.

#### Chromosomal genes

Four antigens (b*mpA*, *flaB*, *oms66*, P83-100) and eight housekeeping genes from the MLST scheme (*clpA*, *clpX*, *nifS*, *pepX*, *pyrG*, *recG*, *rplB*, *uvrA*).

**Ribosomal markers (*rrs*-*rrlA*** **non-coding region)**: Variation in the 16–23 S intergenic spacer was characterized at three levels: (i) IGS typing (the full region), (ii) RSP alleles (ribosomal spacer patterns), and (iii) RST groups (three categories based on RFLP patterns).

Comparisons were also made with 24 reference *B. burgdorferi* s.s. genomes available in GenBank (Table S2).

### Phylogenetic analysis

To test the validity of concatenating multiple genes, we used 13 chromosomal loci, including four chromosomal antigens (*bmpA*, *flaB*, *oms66*, P83-100), eight housekeeping genes (*clpA*, *clpX*, *nifS*, *pepX*, *pyrG*, *recG*, *rplB*, *uvrA*), and the 16–23 S intergenic spacer marker. we applied topology tests (Approximately Unbiased [AU], Shimodaira–Hasegawa [SH], Kishino–Hasegawa [KH]) and Robinson-Foulds (RF) distance comparisons using IQ-TREE v2.1.2^36^. Both a partitioned model (gene-specific evolutionary rates) and a concatenated model (single evolutionary rate) were evaluated.

These tests ensured that concatenation did not introduce significant bias and that the phylogenetic approach accurately captured evolutionary relationships. Maximum likelihood (ML) phylogenetic trees were then constructed using MEGA version 5^37^, with alignments performed in MAFFT tool (v7.450)^38,39^ provided in Geneious prime version 23.1.1. Concatenated core genome sequences (chromosomal antigens, housekeeping genes, 16–23 S marker) and individual accessory genome sequences were analyzed.

Congruence between core and accessory genome trees was assessed using a binary scoring system for monophyly and haplotype correspondence, summarized for each plasmid gene and classification scheme (MLST, *ospC* MGs, IGS, RSP, RST), as opposed to using more conventional metrics such as Robinson-Foulds (RF) distance^40–42^. This decision was driven by the fact that accessory genome trees often do not contain the same set of taxa as the core genome tree (i.e., certain taxa lacked the presence of some plasmid genes). To assess the congruence between the core genome tree and accessory genome trees, we evaluate the monophyly and haplotype correspondence of groups in the core genome tree with the same groups in the accessory genome trees. Specifically, for each monophyletic group defined in the core genome tree, we visually inspected whether that group remained monophyletic in the accessory genome tree (i.e., all members of the group cluster together); and corresponded to the same haplotype (i.e., the group retains its genetic identity) in the accessory genome tree.

For each accessory gene, a binary score was assigned for each group. A score of 1 was given if the group remained monophyletic and corresponded to the same haplotype in the accessory genome tree. A score of 0 was assigned if the group was not monophyletic or did not correspond to the same haplotype.

These scores were summarized in a table for each classification method (MLST, *ospC* major groups, IGS, RSP, RST), and the congruence score for each plasmid gene was calculated as the percentage of congruent groups (i.e., number of groups scored as 1) relative to the total number of groups. This allowed us to assess how well the phylogenetic relationships derived from accessory genome trees aligned with those from the core genome.

### Multi-locus sequence typing (MLST) and geographic distribution analysis

To validate geographic distribution, MLST data were retrieved from PubMLST.org (accessed December 2024). Only entries with complete metadata (host, tick species, geographic location) were included. MLST profiles were cross-referenced with publicly available WGS data. Reported geographic distributions were mapped to study regions, and concordance with phylogenetic clusters was qualitatively assessed.

### Recombination versus mutation analysis

To further investigate the evolutionary dynamics of core and accessory genomes, ClonalFrameML version 3^43^ was used to estimate recombination (R) versus mutation (θ) rates for the core genome and each gene. Higher R/θ values indicate that recombination played a more prominent role in the evolution of these genes compared to mutation-driven divergence.

### Statistical analysis

To explore the relationships between core and accessory genomes and assess population structure, we conducted a series of statistical analyses encompassing correlation testing, clonality measures, and network-based modularity, including:

#### Correlation analysis

Spearman correlation coefficients were calculated between plasmid and core genes based on similarity to the B31 reference genome, to evaluate whether these markers share similar evolutionary trajectories. Positive correlations between core and accessory genes suggest that the genes have followed similar evolutionary trajectories with respect to selection and recombination^44,45^.

#### Hierarchical clustering

Performed using DATAtab online tool^46^ to assess clade divergence under recombination/mutation, providing insights into how genomic processes shape phylogenetic stability.

#### Clonality assessment

goeBURST analysis in Phyloviz v2^47^ identified clonal complexes via single-locus variants (SLVs). Clonality ratios (complexes vs. singletons) were compared between three regions (Manitoba, Ontario, and Nova Scotia), with a Mann–Whitney U test^48,49^ and Cliff’s Delta^50^ applied to quantify differences and effect sizes, thereby testing for geographic variation in clonality. These analyses were conducted in Python using the *scipy.stats*^51^ and *effectsize*^52^ libraries.

#### Network analysis

Conducted in Gephi v0.10.1^53^ within three geographic regions (ON, MB, and NS) to detect modularity (Q index) in genetic clustering, allowing assessment of how strains form cohesive subpopulations based on sequence similarity to reference genomes (Table S2). Genetic similarity was assessed using BLASTn, and only high-confidence alignments with ≥ 99% sequence identity to reference genomes were retained to ensure meaningful homology and minimize spurious connections.

## Results

### Genetic diversity and representativeness of the dataset

Our dataset of 64 whole-genome sequences captures a broad spectrum of *B. burgdorferi* s.s. genetic diversity within Canada. It includes 33 sequence types (STs), representing over one-third of the 90 known STs in the country, covering major genomic lineages circulating in different regions. Additionally, the dataset incorporates 22 *ospC* major groups and subgroups (out of 32 described in Canada), along with 10 intergenic spacer (IGS) types, 12 ribosomal spacer patterns (RSPs), and 3 restriction site types (RSTs). All samples were collected in 2016, minimizing potential temporal bias. While our aim was to capture the overall genetic diversity present in Canada, the dataset was not designed to exhaustively represent the full diversity within each individual region.

### Comparative phylogenetic analysis of partitioned and concatenated evolutionary models

**Fig. 1:**
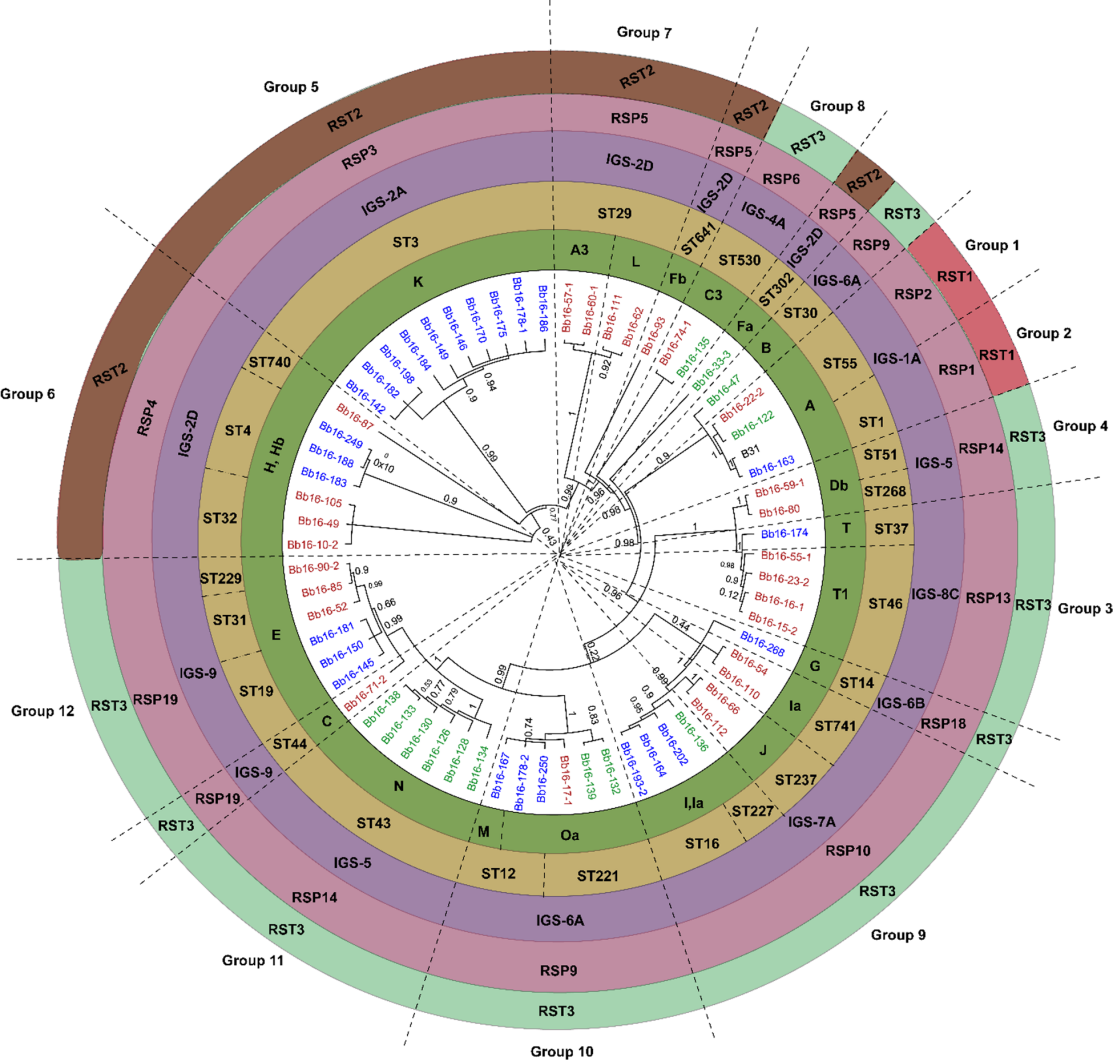
Global maximum likelihood phylogenetic tree of concatenated nucleotide sequences from 13 chromosomal markers of *B. burgdorferi* sensu stricto (*BmpA*, *FlaB*, *oms66*, P83-100, *clpA*, *clpX*, *nifS*, *pepX*, *pyrG*, *recG*, *rplB*, *uvrA*, and the 16–23 S intergenic spacer). Isolates are color‑coded by geographic region: Manitoba (MB, blue), Ontario (ON, green), and Nova Scotia (NS, brown). Colored circle bands indicate genetic markers: *ospC* major groups (bold green), ribosomal sequence types (RST1, red; RST2, brown; RST3, light green), multilocus sequence types (STs, yellow), intergenic spacer (IGS) subtypes (purple), and ribosomal spacer patterns (RSPs, pink). Twelve numbered clades represent well‑supported phylogenetic groups and isolates not clustering within these monophyletic groups were annotated as singletons.

To evaluate the impact of different evolutionary models on phylogenetic inference, we compared partitioned and concatenated models of all chromosomal markers (*BmpA*, *FlaB*, *oms66*, P83-100, *clpA*, *clpX*, *nifS*, *pepX*, *pyrG*, *recG*, *rplB*, *uvrA* and *rrs*-*rrlA*) using IQ-TREE (Fig. S1 and S2). The phylogenetic inference using partitioned and concatenated evolutionary models revealed identical tree topologies, with only minor differences in node support values (Fig. S1, S2). Notably, the only topological difference was observed when comparing the concatenated GTR model (Fig. S2) to the Jukes-Cantor (JC) model (Fig. 1): in the JC tree, Groups 1 and 2 formed a single monophyletic cluster, whereas in the GTR tree, they appeared as two distinct, well-supported clades. This difference arises because the JC model assumes equal substitution rates among all nucleotide changes, making it less sensitive to subtle differences between lineages. As a result, the JC model groups closely related but distinct clades (Groups 1 and 2) into a single cluster with internal substructure. In contrast, the GTR model accounts for variable substitution rates across sites (i.e., it still allows different substitution rates between nucleotides), allowing finer resolution and clearer separation of these groups into distinct clades with stronger statistical support.

Our results (Table S3) indicate that the Robinson-Foulds (RF) distance between the partitioned and concatenated models was minimal (RF distance = 10, normalized RF distance = 0.0769), suggesting strong topological consistency between both approaches. When benchmarked against the core genome phylogeny, the concatenated model achieved a lower normalized RF distance (0.2769) than the partitioned model (0.3385), indicating higher congruence with the core genome. Log-likelihood values were nearly identical between models (− 22557.732 versus − 22557.785), with ΔlogL = 0.052. However, likelihood-based tests - Shimodaira-Hasegawa (p-SH = 1.0 versus 0.0231), Kishino-Hasegawa (p-KH = 0.974 versus 0.0259), and approximate unbiased (p-AU = 0.976 versus 0.0235), strongly favored the concatenated model and statistically rejected the partitioned approach.

Taken together, these results support the validity of concatenation for phylogenetic reconstruction in *B. burgdorferi* s.s., demonstrating that a single evolutionary model across all genes (i.e., chromosomal genes) provides robust and reliable topologies comparable to partitioned approaches, while improving computational efficiency.

### Phylogenetic tree comparison and congruence analysis

The core genome phylogenetic tree constructed in MEGA 5.2.2^37^ with the Jukes-Cantor (JC) model revealed that concatenating sequences of *bmpA*, *flaB*, *oms66*, P83-100, eight housekeeping genes (*clpA*, *clpX*, *nifS*, *pepX*, *pyrG*, *recG*, *rplB*, *uvrA*), and the 16–23 S chromosomal marker, divided 59 of the 64 *B. burgdorferi* s.s. strains into 12 well-supported monophyletic groups that were also classified based on MLST, IGS, RSP, and RST markers. Group delineation was based either on the most recent common ancestor (MRCA) with high bootstrap support (≥ 0.95) or, in cases where a larger clade contained well-resolved subclades, on the consistency of those subclades with one or more independent typing methods (MLST, *ospC* MG, IGS, RSP, RST). The other five strains were singletons (Fig. 1). While higher-order branching patterns sometimes differed between core genome and plasmid-gene trees, the internal monophyly of each group was preserved across datasets. Branch lengths in the phylogenetic tree are proportional to nucleotide divergence, allowing inference of relative evolutionary distances among and within clades. These phylogenetic groups and their respective geographic distributions, based on the sequence types (STs) reported in the pubmlst.org database (Table S4), are as follows:

• Group 1 (ST55, IGS-1 A, RSP2, RST1): ST55 is found in the Midwestern USA (Minnesota, Wisconsin) and south central Canada (Manitoba, Ontario).
• Group 2 (ST1, IGS-1 A, RSP1, RST1): ST1 is predominantly distributed in the Northeastern USA (Connecticut, Massachusetts, Maine, New Hampshire, New York, Pennsylvania, Rhode Island, Virginia, Vermont) and central and southeastern Canada (Ontario, Quebec, and the Maritimes).
• Group 3 (ST46, IGS-8 C, RSP13, RST3): ST46 is found in the Midwestern USA (Wisconsin, Minnesota) and south central Canada (Ontario, Manitoba).
• Group 4 (ST51, ST268, IGS-5, RSP14): STs 51 and 268 are found in the Northeastern and upper Midwestern USA (Minnesota, Wisconsin, New York) and central and western Canada (Manitoba, British Columbia).
• Group 5 (ST3, IGS-2 A, RSP3, RST2): ST3 is primarily found in the Northeastern USA (Massachusetts, New York, Connecticut, Vermont, Virginia, Rhode Island, New Jersey) and southeastern Canada (Maritimes, Ontario, Quebec).
• Group 6 (ST4, ST32, ST740, IGS-2D, RSP4, RST2): STs 4, 32 and 740 are distributed across the Northeastern and Midwestern USA (Connecticut, Illinois, Massachusetts, Maine, Michigan, Minnesota, New Jersey, New York, Pennsylvania, Rhode Island, Wisconsin) and southern Canada (Manitoba, Ontario, Quebec, and the Maritimes).
• Group 7 (ST29, IGS-2D, RSP5, RST2): ST29 is found in the Northeastern and Midwestern USA (Connecticut, Illinois, Minnesota, Wisconsin) and across southern Canada (British Columbia, Manitoba, Ontario, Quebec).
• Group 8 (ST530, IGS-2D, RSP5, RST2): ST530 was identified in the upper Midwestern USA (Wisconsin, Minnesota) and south central Canada (Manitoba, Northwestern Ontario).
• Group 9 (ST16, ST227, ST237, ST741, IGS-7 A, RSP10, RST3): STs 16, 227, 237 and 741 are distributed across the Northeastern and upper Midwestern USA (Connecticut, Massachusetts, New York, Rhode Island, Wisconsin) and across southern Canada (British Columbia, Manitoba, Ontario, Quebec, and the Maritimes).
• Group 10 (ST12, ST221, IGS-6 A, RSP9, RST3): STs 12 and 221 are found in northeastern and upper Midwestern USA (Connecticut, Massachusetts, New York, Rhode Island, Wisconsin, Michigan, Illinois) and across southern Canada (British Columbia, Manitoba, Ontario, Quebec, and the Maritimes).
• Group 11 (ST43, IGS-5, RSP14, RST3): ST43 is found in the upper Midwestern USA (Wisconsin, Minnesota) and western and south central Canada (British Columbia, Manitoba, Northwestern Ontario).
• Group 12 (ST19, ST31, ST229, IGS9, RSP19, RST3): STs 19, 31 and 229 are distributed across the Northeastern and upper Midwestern USA (Connecticut, New York, US Midwest) and south central and southeastern Canada (Manitoba, Ontario, Quebec, and the Maritimes).

Notably, several core phylogenetic groups correspond to unique combinations of ST, IGS, and RSP types, such as Group 1 (ST55, IGS-1 A, RSP2, RST1), Group 2 (ST1, IGS-1 A, RSP1, RST1), and Group 5 (ST3, IGS-2 A, RSP3, RST3) (Table S3). This classification provides a framework for understanding the evolutionary relationships of *B. burgdorferi* s.s. strains. Seven strains had unique combinations of MLST, IGS, RSP, and RST types (Fig. 1).

### Congruence across classification methods

To assess the consistency between core and plasmid-encoded genes, congruence was calculated between these core genome groups and the plasmid gene trees (Figures S3–S13) for *ospC*, *dbpA*, *dbpB*, *oms28*, *ospA*, *ospB*, *ospD*, Fibronectin P35, P37, P45-13 and C6 peptide of *vlsE1* using different classification methods (Tables S5-S9). The tables provide a quantitative summary of the visual comparisons shown in Figures S3–S13, converting tree‑based congruence into a binary scoring system to facilitate direct comparison.

#### MLST classification

The MLST classification method consistently exhibited high congruence with the core genome tree across most phylogenetic groups. Notably, Group 1 (ST55), Group 2 (ST1), Group 3 (ST46), and Group 8 (ST530) achieved a high congruence (91%-100%) across all genes, indicating strong evolutionary coherence (Table S5). Fibronectin P35 showed high congruence (91%), indicating its stable alignment with the core genome. Similarly, *dpbA*,* dpbB*, and *ospC* also demonstrated high congruence (75%), reflecting their phylogenetic alignment with core genome markers. Conversely, *ospA* exhibited the lowest congruence (27%).

#### OspC major group classification

The major *ospC* grouping was variably congruent with the core genome and other plasmid markers. The C6 peptide of VlsE1 achieved the highest congruence (100%), followed by *ospC* (75%) and P45-13 (73%), indicating strong alignment between these genes and the core genome. However, Group 1 (ST55, *ospC* A) showed a lower congruence score (55%) compared to Group 2 (ST1, *ospC* A) at 73%, indicating differences in phylogenetic consistency between these groups. Genes like *ospA* (27%) and oms28 (42%) had lower congruence with *ospC* (Table S6).

#### IGS classification

The IGS classification method was poorly congruent with other markers. The C6 peptide showed the highest congruence with IGS (67%) across groups, particularly in Groups 3, 5, 8, 10 and 12. Other genes, such as Fibronectin P35 (18%) and *ospA* (18%), exhibited low congruence with core genome (Table S7).

#### RSP classification

The RSP classification method demonstrated moderate to high congruence with other markers. Groups 1, 3, and 8 RSP were > 78% congruent with *ospD*, C6, and P37, but RSP classification was poorly congruent with the *ospA* (36%) (Table S8).

#### RST classification

The RST classification method displayed the lowest overall congruence scores. *dpbA*, *dpbB*, *oms28*, *ospC*, and *ospD* showed minimal congruence with RST classification (respectively 17%, 33%, 8%, 17%, 25%), while others like *ospA*, *ospB*, Fibronectin P35, P45-13 and P37 exhibited no congruence (0%) (Table S9).

#### Group-level insights and general observations

Groups 1, 2, 3, and 8 showed the highest congruence scores across multiple classification methods, suggesting they form stable, monophyletic clades. Notably, Group 8 (*ospC* allele C3) exhibited high evolutionary stability across genes - but this is a very small group (only 2 strains). Conversely, Group 2 (ST1, *ospC* A) showed lower congruence compared to Group 1 (ST55, *ospC* allele A) (Fig. S3-S13).

Across all classification methods, MLST and *ospC* major groups displayed the highest overall congruence. In contrast, RST classification consistently showed the lowest congruence.

#### General insights across classification methods

The MLST and *ospC* major group classification methods demonstrated the highest overall congruence with the core genome. Genes such as, C6 peptide of vlsE1, and *dbpA* consistently showed high congruence across multiple methods (Fig. S3-S13). Conversely, *ospA* and P45-13 consistently exhibited lower congruence across all classification methods. The RST classification method demonstrated the lowest congruence across all genes.

### Recombination versus mutation analysis

To understand the evolutionary forces shaping *B. burgdorferi* s.s. populations, we compared the impact of recombination versus mutation across the core genome and several plasmid-encoded genes using ClonalFrameML. The R/θ ratio, representing the relative contribution of recombination to mutation, revealed distinct patterns across different genomic regions. The core genome exhibited a moderately high R/θ ratio of 1.50 (Table 1), indicating that recombination plays a significant role in its evolution. However, while mutation contributes to genetic variation, its effects are generally more constrained compared to recombination, which can introduce larger-scale genomic changes. This relative constraint may help preserve the integrity of essential chromosomal regions.

**Table 1:** Estimates of the recombination-to-mutation ratio (R/θ) from clonalframeml for the core genome (*BmpA*, *FlaB*, *oms66*, P83-100, and eight MLST housekeeping genes) and 11 plasmid-encoded genes of *Borrelia burgdorferi* sensu stricto. Higher values indicate a greater contribution of recombination relative to mutation in shaping genetic diversity.

Gene/Genome region	*R*/θ Ratio	Posterior mean	Posterior variance
Core Genome	1.5	1.500	0.010
*dbpA*	2.32	2.319	0.179
*dbpB*	0.16	0.160	0.015
*ospA*	0.1	0.099	0.008
*ospB*	0.08	0.078	0.006
*ospC*	4.25	4.251	0.345
*ospD*	0.1	0.098	0.009
*oms28*	0.18	2.07	11.79
Fibronectin P35	0.42	0.418	0.030
P37	0.2	0.197	0.019
P45-13	0.08	0.0053	1.457
C6	0.23	0.228	0.023

Among the plasmid‑encoded antigens, recombination rates varied substantially: *ospC* showed the highest recombination relative to mutation (R/θ = 4.25), whereas *ospA* and P45‑13 exhibited markedly lower ratios, and *ospB* showed intermediate values (Table 1). These findings highlight the differential evolutionary pressures acting on major surface antigens.

### Statistical analysis

*Core genome and accessory gene relationships*: To further investigate the degree of evolutionary consistency between core genome and accessory genes in *B. burgdorferi* s.s., we calculated the distance (as measure by % nucleotide divergence) of each gene, in every strain, to the homologue on the reference genome B31. We then checked for the strength of correlations in these distances between different pairs of genes (Table 2). Significant positive correlations were found between the C6 peptide and chromosomal genes: *bmpA* (*r* = 0.76, R² = 0.58, *p* < 0.001), *flaB* (*r* = 0.63, R² = 0.4, *p* < 0.001), and sequence types (ST) (*r* = 0.81, R² = 0.65, *p* < 0.001), IGS (*r* = 0.81, R² = 0.65, *p* < 0.001), RSP (*r* = 0.54, R² = 0.3, *p* = 0.003), and RST (*r* = 0.57, R² = 0.32, *p* = 0.002) (Table 2). Similarly, *dbpA* showed moderate positive correlations with *bmpA* (*r* = 0.6, R² = 0.35, *p* < 0.001), *flaB* (*r* = 0.55, R² = 0.31, *p* < 0.001), and ST (*r* = 0.59, R² = 0.34, *p* < 0.001). Negative correlations revealed potential evolutionary divergence. *ospC* showed negative correlations with *oms66* (*r* = -0.39, R² = 0.15, *p* = 0.002) and P83-100 (*r* = -0.35, R² = 0.12, *p* = 0.005) (Table 2). Similarly, *oms28* was negatively correlated with *flaB* (*r* = -0.3, R² = 0.09, *p* = 0.018).

**Table 2:** Spearman correlation analysis of similarity values to the *Borrelia burgdorferi* sensu stricto B31 reference genome for 11 plasmid-encoded genes and 8 core chromosomal genomic markers across 64 Canadian strains. Reported statistics include the coefficient of determination (R²), correlation coefficient (rho), and associated p-values (p) for each gene pair comparison.

Plasmid/core genomes	*R*² (coefficient of determination)	Spearman *r* correlation	*P*-value
C6 and *BmpA*	0.58	0.76	< 0.001
C6 and *FlaB*	0.4	0.63	< 0.001
C6 and *oms66*	0	0	0.996
C6 and P83-100	0.02	-0.13	0.501
C6 and IGS	0.65	0.81	< 0.001
C6 and RSP	0.3	0.54	0.003
C6 and RST	0.32	0.57	0.002
C6 and ST	0.65	0.81	< 0.001
*dbpA* and *BmpA*	0.35	0.6	< 0.001
*dbpA* and *FlaB*	0.31	0.55	< 0.001
*dbpA* and oms66	0.01	0.11	0.393
*dbpA* and P83-100	0	-0.05	0.717
*dbpA* and IGS	0.01	0.08	0.557
*dbpA* and RSP	0.1	-0.31	0.012
*dbpA* and RST	0.05	-0.22	0.077
*dbpA* and ST	0.34	0.59	< 0.001
*dbpB* and *BmpA*	0.04	0.2	0.12
*dbpB* and *FlaB*	0.03	0.18	0.166
*dbpB* and *oms66*	0	-0.04	0.742
*dbpB* and P83-100	0.01	-0.1	0.421
*dbpB* and IGS	0	-0.01	0.96
*dbpB* and RSP	0.01	0.11	0.398
*dbpB* and RST	0.03	0.18	0.167
*dbpB* and ST	0.16	0.4	0.001
*ospC* and *BmpA*	0.05	0.22	0.086
*ospC* and *FlaB*	0.1	0.32	0.01
*ospC* and *oms66*	0.15	-0.39	0.002
*ospC* and P83-100	0.12	-0.35	0.005
*ospC* and IGS	0.11	0.32	0.01
*ospC* and RSP	0.22	0.47	< 0.001
*ospC* and RST	0.19	0.44	< 0.001
*ospC* and ST	0	0.05	0.705
*Oms28* and *BmpA*	0	0.05	0.687
*Oms28* and *FlaB*	0.09	-0.3	0.018
*Oms28* and *oms66*	0.16	0.4	0.001
*Oms28* and P83-100	0.01	0.09	0.487
*Oms28* and IGS	0.01	-0.11	0.373
*Oms28* and RSP	0	-0.04	0.764
*Oms28* and RST	0	-0.02	0.861
*Oms28* and ST	0.01	0.1	0.44
*ospA* and *BmpA*	0	-0.05	0.766
*ospA* and *FlaB*	0	-0.03	0.842
*ospA* and *oms66*	0.02	-0.13	0.451
*ospA* and P83-100	0.06	0.24	0.162
*ospA* and IGS	0	-0.01	0.973
*ospA* and RSP	0.01	-0.09	0.618
*ospA* and RST	0	-0.01	0.939
*ospA* and ST	0.11	0.34	0.045
*ospB* and *BmpA*	0.13	0.37	0.005
*ospB* and *FlaB*	0.01	0.07	0.59
*ospB* and *oms66*	0.08	0.28	0.033
*ospB* and P83-100	0.02	-0.14	0.291
*ospB* and IGS	0.01	-0.12	0.384
*ospB* and RSP	0.03	-0.18	0.185
*ospB* and RST	0.04	-0.21	0.123
*ospB* and ST	0.1	0.32	0.015
*ospD* and *BmpA*	0.13	0.37	0.003
*ospD* and *FlaB*	0.32	0.57	< 0.001
*ospD* and *oms66*	0.02	0.15	0.232
*ospD* and P83-100	0.03	-0.16	0.214
*ospD* and IGS	0	-0.02	0.847
*ospD* and RSP	0.02	-0.15	0.232
*ospD* and RST	0.07	-0.27	0.032
*ospD* and ST	0.35	0.59	< 0.001
Fibronectin P35 and *BmpA*	0.1	-0.32	0.015
Fibronectin P35 and *FlaB*	0.17	-0.41	0.001
Fibronectin P35 and *oms66*	0.21	0.46	< 0.001
Fibronectin P35 and P83-100	0.25	0.5	< 0.001
Fibronectin P35 and IGS	0.09	-0.3	0.023
Fibronectin P35 and RSP	0.01	-0.09	0.498
Fibronectin P35 and RST	0	-0.04	0.778
Fibronectin P35 and ST	0.02	-0.12	0.364
p37 and *BmpA*	0.66	0.82	< 0.001
p37 and *FlaB*	0.32	0.57	0.014
p37 and *oms66*	0	-0.01	0.956
p37 and P83-100	0.02	0.14	0.584
p37 and IGS	0.13	0.36	0.141
p37 and RSP	0.5	0.71	0.001
p37 and RST	0.51	0.71	0.001
p37 and ST	0.64	0.8	< 0.001
P45-13 and *BmpA*	0.06	-0.24	0.101
P45-13 and *FlaB*	0.01	-0.12	0.417
P45-13 and *oms66*	0.02	-0.15	0.295
P45-13 and P83-100	0.01	0.1	0.508
P45-13 and IGS	0.01	-0.08	0.607
P45-13 and RSP	0.02	0.16	0.281
P45-13 and RST	0.03	0.17	0.243
P45-13 and ST	0.07	-0.27	0.062

#### Hierarchical clustering analysis

To further examine how different genes reveal consistent patterns of sequence similarity to the B31 reference genome, hierarchical clustering analysis was performed using Euclidean distance and single-linkage methods. This analysis allowed us to visualize how the degree of similarity to the B31 reference genome translated into phylogenetic relationships among the core and accessory genomes. Strains carrying *ospC* alleles I (i.e., including Ia subtype) and K consistently formed monophyletic clades in both core and accessory genome trees, as confirmed by maximum likelihood (ML) phylogenetic trees with high bootstrap support values (1 for *ospC* I-Ia and 0.99 for *ospC* K) (Fig. S3). These clusters aligned with the positive correlations observed between *ospC* and markers like RSP (*r* = 0.47, R^2^ = 0.22, *p* < 0.001) and RST (*r* = 0.44, R^2^ = 0.19, *p* = 0.001) (Table 2).

**Fig. 2:**
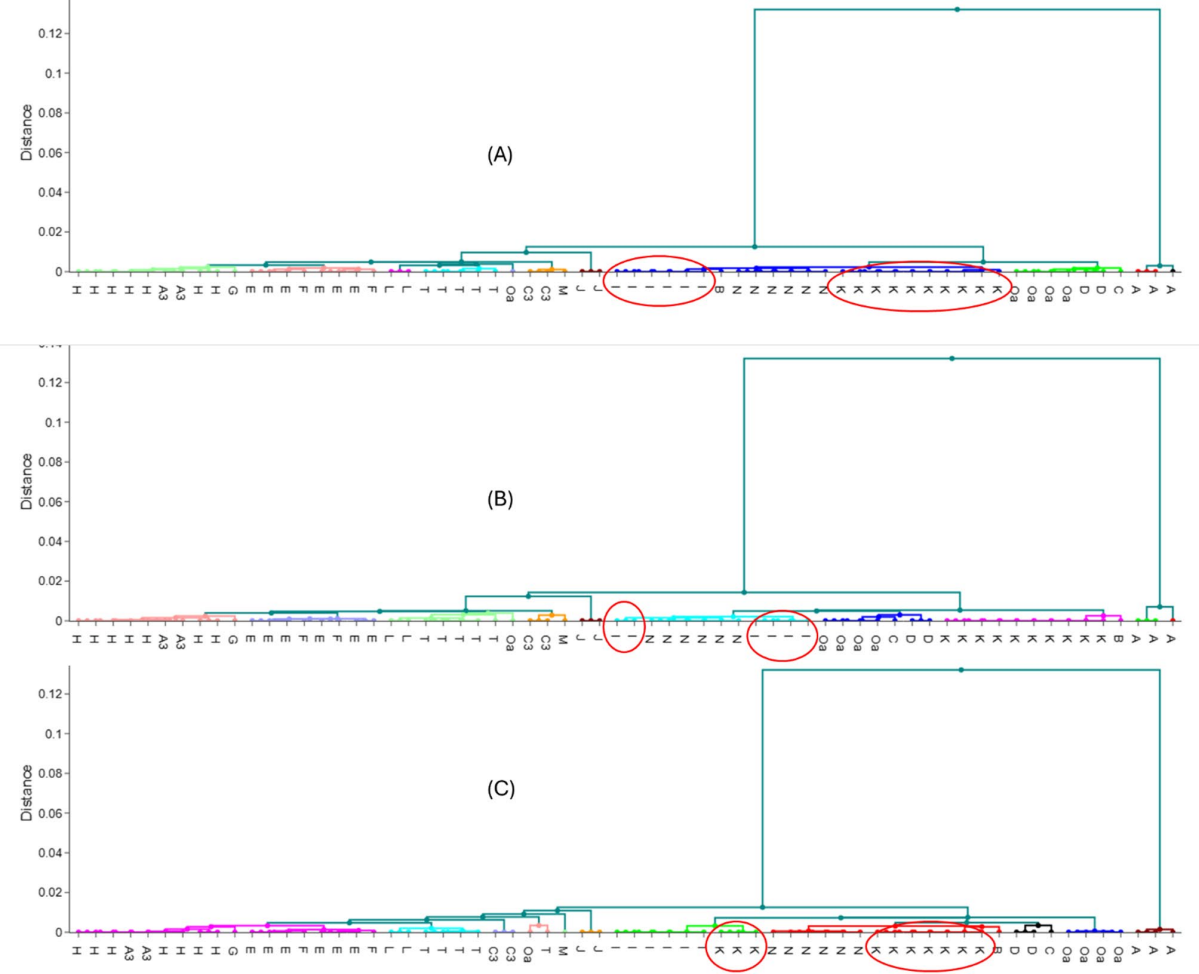
Hierarchical dendrograms illustrating relationships between the plasmid gene *ospC* and selected chromosomal genomic markers in *Borrelia burgdorferi* sensu stricto. Panels show: (**A**) *ospC* vs. *FlaB* (positive correlation), (**B**) *ospC* vs. *oms66* (negative correlation), and (**C**) *ospC* vs. P83-100 (negative correlation). The dendrograms include all 64 Canadian strains, with *ospC* molecular genotypes (MGs) labeled. Genotypes influenced by negative correlations are highlighted with red circles. The colors in the dendrogram denote distinct clusters.

However, negative correlations between *ospC* and core proteins such as *oms66* were reflected in phylogenetic splits. The hierarchical clustering analysis revealed that strains carrying *ospC* I-Ia (Fig. 2A and B) were divided into two distinct clusters. A similar pattern was observed with *ospC* K (Fig. 2A and C), where the negative correlation with P83-100 resulted in a phylogenetic split.

#### Degree of clonality and modularity of B. burgdorferi

To assess the degree of clonality in *B. burgdorferi* s.s. populations from different geographic regions (NS, ONMB), we compared the ratio of haplotypes to singletons. Ontario and Manitoba were combined due to the lack of significant statistic differences in their *B. burgdorferi* s.s. population clonality (data not shown). A Mann-Whitney U test revealed a statistically significant difference in clonality, measured as haplotype-to-singleton ratios, between the *B. burgdorferi* s.s. populations in Nova Scotia (NS) and the combined Ontario/Manitoba (ONMB) regions (U = 975, *p* < 0.001) showing that the degree of clonality is higher in NS compared to ONMB (Table 3).

**Table 3:** Results of the Mann–Whitney U test comparing haplotype-to-singleton ratios, based on clonal complexes defined by single-locus variants (SLVs), between *Borrelia burgdorferi* sensu stricto populations in Nova Scotia (NS) and combined Ontario/Manitoba (ONMB) regions. Cliff’s delta values are included to indicate effect size and direction of differences.

Comparison	*n* (NS)	*n* (ONMB)	Median (NS)	Median (ONMB)	Mann-Whitney U	Z-score	Asymptotic *p* (2-tailed)	Effect Size (*r*)	Cliff’s Delta
NS vs. ONMB (Haplotype/Singleton)	25 (20/5)	39 (24/15)	4	1.6	19	-7.78	2.21 × 10⁻¹⁵	0.98	+ 1

Cliff’s Delta had a value of + 1, indicating all clonality ratios in NS were greater than those observed in ONMB, supporting the interpretation that the NS population demonstrates a consistently higher level of clonality relative to ONMB (Table 3).

*Network analysis*: To uncover regional patterns in population structure, we conducted network-based modularity and association analyses across the three regions: Nova Scotia (NS), Ontario (ON), and Manitoba (MB). In this analysis, we treated Ontario and Manitoba separately, unlike in the clonality analysis where they were grouped together. This distinction was made because clonality measures genetic redundancy within a population, whereas modularity assesses the presence of distinct genetic clusters (subpopulations) within a region. Since ON and MB share many strain types, it made sense to group them for clonality analysis. However, for modularity analysis, we observed regional genetic differentiation, with some groups being exclusive to Manitoba (Groups 3, 4, 7) and others to Ontario (Group 11), which warranted separate analyses.

The modularity index (Q) confirmed moderate to strong community structures across all regions. The Nova Scotia (NS) population had the highest modularity (Q = 0.68), indicating well-defined genetic clusters. In contrast, Ontario exhibited the lowest modularity (Q = 0.508), suggesting a more fragmented and mixed population structure, while Manitoba had an intermediate modularity value (Q = 0.634), reflecting moderate population structuring.

**Fig. 3:**
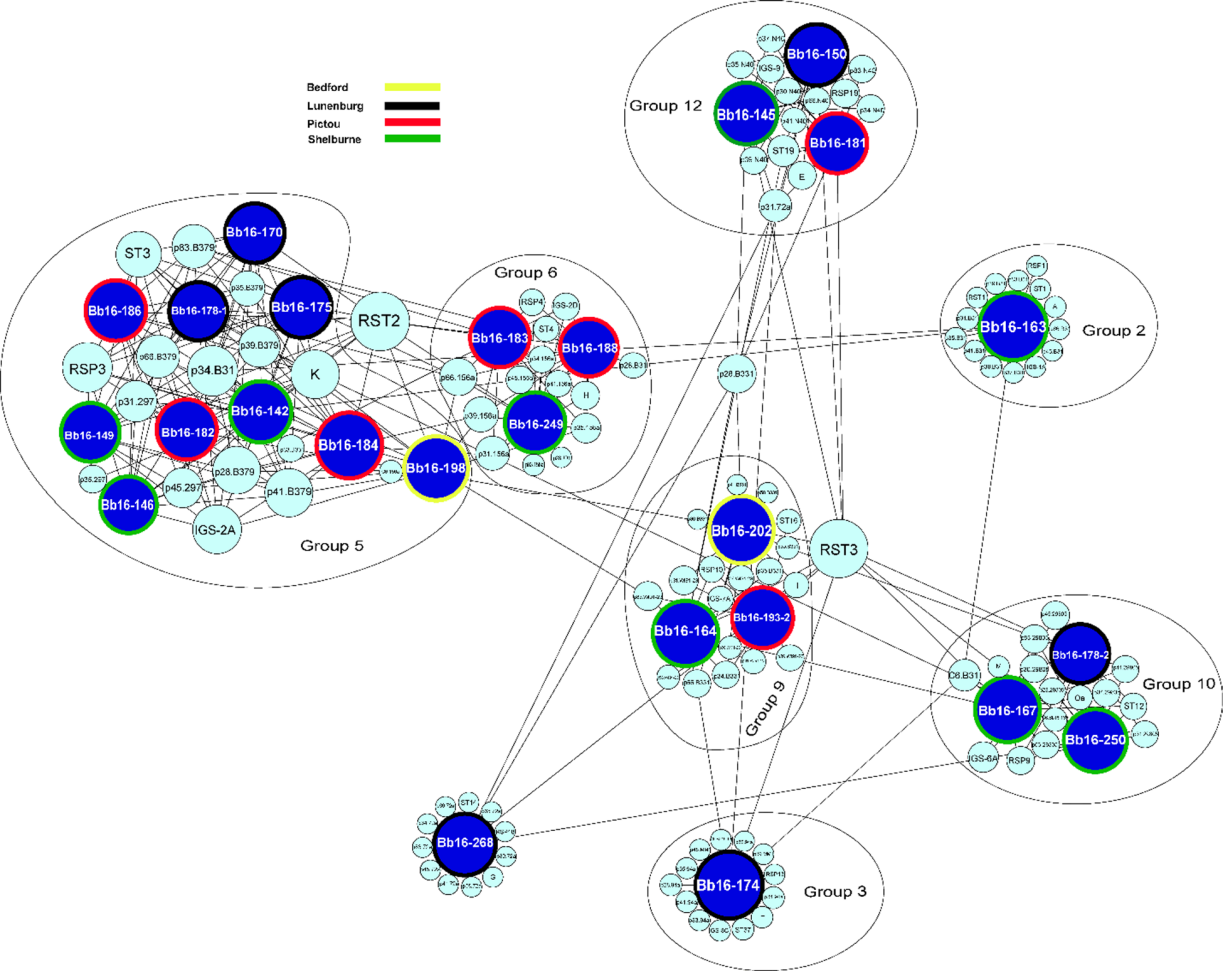
Network graph illustrating genetic relationships among 26 *Borrelia burgdorferi* sensu stricto strains collected in Nova Scotia, Canada. Relationships are based on a comprehensive set of chromosomal and plasmid-encoded genomic markers. Chromosomal markers include *BmpA* (P39), *FlaB* (P41), *oms66* (P66), and P83-100 (P83), together with eight housekeeping genes (*clpA*, *clpX*, *nifS*, *pepX*, *pyrG*, *recG*, *rplB*, *uvrA*) and the C6 peptide of *VlsE1*. Plasmid-encoded markers include *dbpA* (P17), *dbpB* (P18), fibronectin-binding protein (P35), *oms28* (P28), *ospA* (P31), *ospB* (P34), *ospC* (MG), *ospD* (P30), P37, and P45-13 (P45). Strain nodes are color-coded by sampling location: yellow for Bedford, black for Lunenburg, red for Pictou, and green for Shelburne. Genomic marker nodes are shown in cyan. Black edges represent the highest sequence similarity scores, as determined by BLAST analysis, indicating genetic connections among strains based on shared markers.

**Fig. 4:**
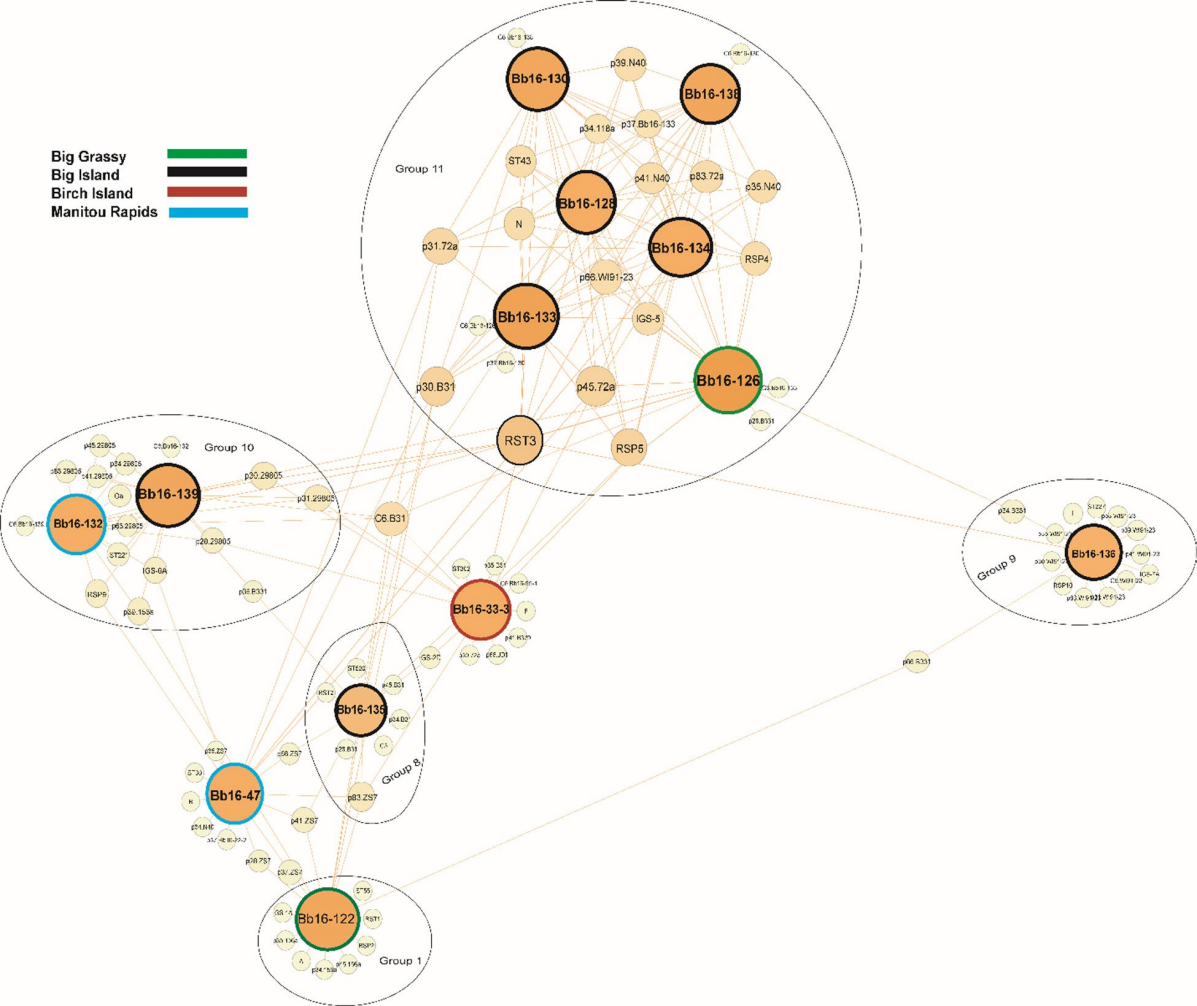
Network graph illustrating genetic relationships among 26 *Borrelia burgdorferi* sensu stricto strains collected in Ontario, Canada. Relationships are based on a comprehensive set of chromosomal and plasmid-encoded genomic markers. Chromosomal markers include *BmpA* (P39), *FlaB* (P41), *oms66* (P66), and P83-100 (P83), together with eight housekeeping genes (*clpA*, *clpX*, *nifS*, *pepX*, *pyrG*, *recG*, *rplB*, *uvrA*) and the C6 peptide of *VlsE1*. Plasmid-encoded markers include *dbpA* (P17), *dbpB* (P18), fibronectin-binding protein (P35), *oms28* (P28), *ospA* (P31), *ospB* (P34), *ospC* (MG), *ospD* (P30), P37, and P45-13 (P45). Strain nodes are color-coded by sampling location: green for Big Grassy, black for Big Island, brown for Birch Island, and blue for Manitou Rapids. Genomic marker nodes are shown in yellow. Orange edges represent the highest sequence similarity scores, as determined by BLAST analysis, indicating genetic connections among strains based on shared markers.

**Fig. 5:**
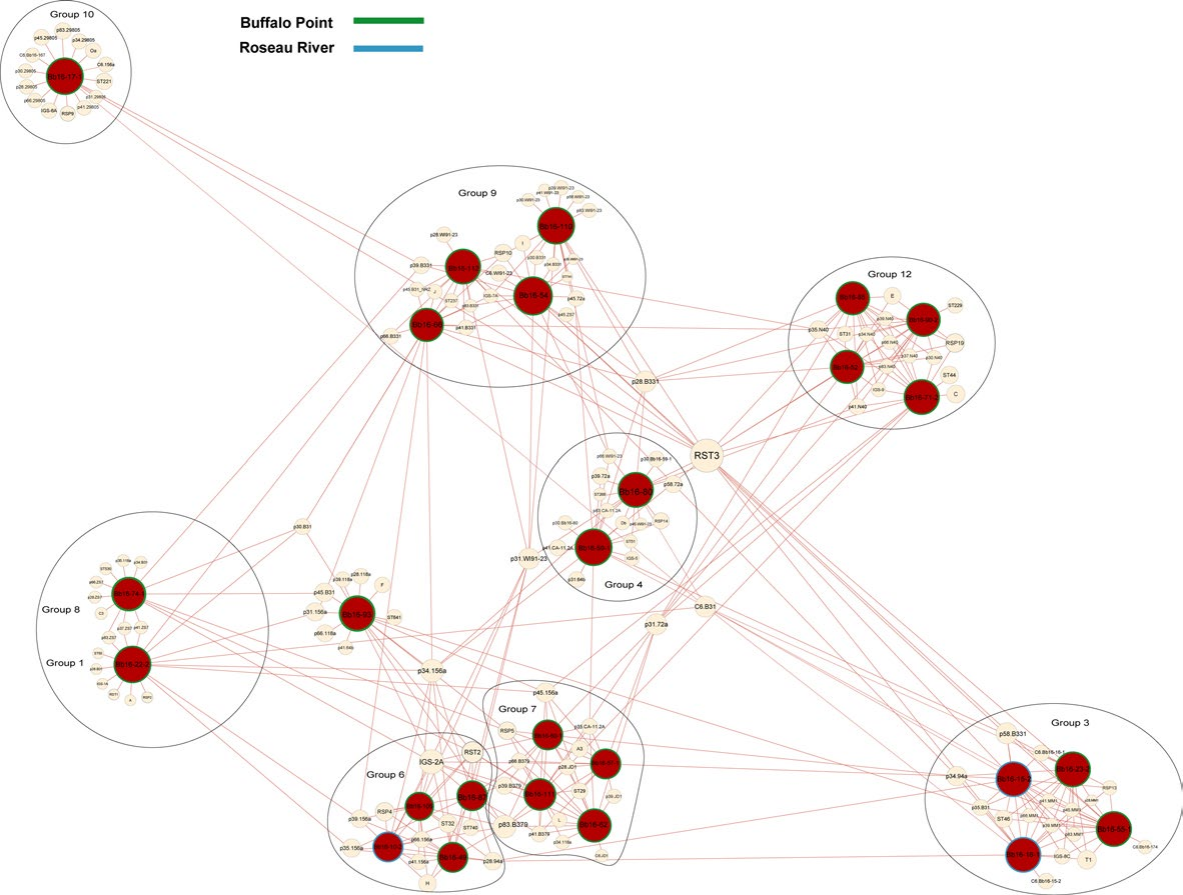
Network graph illustrating genetic relationships among 26 *Borrelia burgdorferi* sensu stricto strains collected in Manitoba, Canada. Relationships are based on a comprehensive set of chromosomal and plasmid-encoded genomic markers. Chromosomal markers include *BmpA* (P39), *FlaB* (P41), *oms66* (P66), and P83-100 (P83), together with eight housekeeping genes (*clpA*, *clpX*, *nifS*, *pepX*, *pyrG*, *recG*, *rplB*, *uvrA*) and the C6 peptide of *VlsE1*. Plasmid-encoded markers include *dbpA* (P17), *dbpB* (P18), fibronectin-binding protein (P35), *oms28* (P28), *ospA* (P31), *ospB* (P34), *ospC* (MG), *ospD* (P30), P37, and P45-13 (P45). Strain nodes are color-coded by sampling location: green for Buffalo Point and blue for Roseau River. Genomic marker nodes are shown in peach. Brown edges represent the highest sequence similarity scores, as determined by BLAST analysis, indicating genetic connections among strains based on shared markers.

Beyond clustering, our network analysis highlighted clear genetic associations between *B. burgdorferi* s.s. strains in Nova Scotia, Ontario, and Manitoba and their respective reference strains, alongside shared genomic markers. Seven of the 12 monophyletic groups identified in Nova Scotia were closely related to reference strains, suggesting that these strains share a common evolutionary history while also displaying regional genetic divergence (Fig. 3). For example, Group 2 was genetically identical to the B31 reference strain, suggesting a direct lineage connection, while Group 5 showed similarities with B379 and 297, and Group 6 was closely related to the 156a strain. Group 12 exhibited genetic proximity to N40, reflecting a shared evolutionary background. The network analysis revealed distinct genetic associations between *B. burgdorferi* s.s. strains and reference strains across different regions, providing insights beyond core genome-based phylogenies. In Ontario, Groups 1, 8, 9, 10, and 11 aligned closely with reference strains 156a, ZS7, B331, 29,805, WI91-23, and N40 (Fig. 4), supported by genetic markers such as IGS-1 A (Group 1) and IGS-2D/RSP5 (Group 8). In Manitoba, nine of the twelve groups displayed distinct relationships, with Groups 6 and 7 associating with 94a, JD1, and 118a, while Groups 4 and 12 aligned with CA11-2 A and WI91-23 (Fig. 5). In Nova Scotia, Groups 2 and 5 were exclusive to this region, with Bb163 from Group 2 being genetically identical to the B31 reference strain. Notably, the same phylogenetic group sometimes aligned with different reference strains depending on the region; for instance, Group 1 aligned with 156a in Ontario but ZS7, 118a, and B331 in Manitoba. This network-based approach complements phylogenetic trees by incorporating gene-specific alignments, particularly for plasmid genes, which are not fully captured in core genome-based phylogenies.

## Discussion

This study provides a comprehensive analysis of the genetic diversity and evolutionary dynamics of *B. burgdorferi* s.s. across three Canadian regions: Nova Scotia (NS), northwest Ontario (ON), and southeastern Manitoba (MB). Its northward expansion into Canada reflects recent introductions linked to *I. scapularis* range shifts^31,54^. Similar to U.S. populations, Canadian strains show complex region-specific trajectories shaped by multiple introductions, recombination, and local ecology^29,55–57^. Unlike *B. bavariensis*, which experienced a strong bottleneck^58^, *B. burgdorferi* s.s. in Canada exhibits a more complex population structure.

Our first objective was to explore the phylogenetic consistencies between chromosomal and plasmid-borne genes, and then how recombination and mutation contribute to the genetic structure of *B. burgdorferi* s.s. These comparisons inform on the reliability of these markers for typing and are relevant for understanding ecological determinants of diversity.

We identified 12 well-defined groups (excluding five singletons) supported by MLST, *ospC*, RSP, RST, and IGS markers, consistent with whole-genome phylogenies from Canada and the U.S. (e.g., Group 5 corresponds to Tyler et al.’s Clade K; Groups 1–2 reflect their Clade A subdivision)^35^. Statistical tests confirmed that concatenation of markers was appropriate. Interestingly, plasmid-encoded surface proteins and chromosomal genes showed unexpected phylogenetic concordance. This contrasts with European *B. burgdorferi* sensu lato species, where ospC often shows incongruence due to horizontal transfer across species (e.g., Fr-93-1 sharing ospC with *B. finlandensis*^59^. These observations indicate that interspecific introgression is a major evolutionary force shaping *ospC* diversity in Europe. Further study is needed to understand these differences.

### *Borrelia burgdorferi* s.s groups

A literature review revealed consistent associations between genotypes, geography, and host ecology. Groups 7 and 9 occur broadly, while Groups 1, 3, and 8 occur in central regions (southern Canada/upper Midwest), and Groups 2 and 5 are found in the northeast. Group 11 is absent from eastern North America, and Groups 6 and 12 are not found west of the Rocky mountains^16,17,19,20,60–62^. Group 7 is widespread across reservoir hosts^17,19,29,35,62^. Group 2 (ST1) is associated with *Peromyscus leucopus*, and absent from much of central North America^63,64^. Group 6 (often carrying ospC K) is likely specialized for mice but can be reservoired by both *P. maniculatus* and *P. leucopus*^18,61,65^. Group 3 may be associated with chipmunk hosts as suggested by studies that associate *ospC* allele T with this species^11,18,65^.

### Phylogenetic comparisons between core and accessory genomes

Our phylogenetic analysis revealed strong congruence between the core genome and specific plasmid-encoded genes within certain phylogenetic groups. Group 1 (ST55), Group 2 (ST1), Group 3 (ST46), and Group 8 (ST530) demonstrated 100% congruence between core genome markers and multiple plasmid genes. This high congruence aligns with findings from previous studies, which suggest that certain sequence types (STs) of *B. burgdorferi* s.s. exhibit strong genetic coherence due to shared evolutionary constraints and selection pressures acting on both core and accessory genomes^56,66^. The parallel inheritance of core and plasmid genes within these groups suggests that clonal expansion, rather than frequent recombination, plays a major role in shaping the genetic structure of these lineages. This supports previous evidence that recombination (or horizontal plasmid transfer) does not necessarily disrupt the alignment between core and accessory genomes^67^.

Margos et al. (2012) highlighted how geographic boundaries in the United States restrict genetic exchange between northeastern, midwestern, and western *B. burgdorferi* s.s. populations. Geographic barriers, reinforced by ecological factors, limit gene flow across regions. Strain-host associations may further contribute to this genetic isolation, if certain strains preferentially infect specific host species, creating additional ecological barriers to gene flow.

Margos et al. (2012) demonstrated that, geographically distinct strains, such as ST1 and ST55, exhibit unique clonal lineages while still sharing identical accessory genome elements, such as the *ospC* allele (A) Brisson and colleagues (2010) argued that northeastern and midwestern *(B) burgdorferi* s.s. populations share a common ancestor. Our findings support these studies as we identified significant divergence between Group 1 and Group 2, particularly regarding core genomic markers.

This underscores the role of geographic factors, including host and vector ecology^20^, in driving genetic differentiation within *B. burgdorferi* s.s. populations. Similar trends are documented in other bacterial species, such as *Francisella tularensis*^68^ and *Yersinia pestis*^69^, where spatially distinct populations adapt to local hosts, resulting in unique evolutionary trajectories.

### Recombination versus mutation - evolutionary dynamics

To further understand the evolutionary forces shaping *B. burgdorferi* s.s., we evaluated the recombination and mutation rates in core and accessory genomes using ClonalFrameML. The R/θ ratio (recombination-to-mutation) was moderately high for the core genome (1.50), suggesting that recombination plays a significant role in its evolution, though not at a sufficient frequency to erode the phylogenetic signal.

Phylogenetic consistency and variability in recombination rates within the accessory genome can reflect adaptive evolutionary strategies in *B. burgdorferi* s.s. Specifically, *ospC* exhibited high R/θ ratios, and this gene is under diversifying selection driven by host adaptation responses^70^. Notably, *ospA* exhibited the lowest congruence with the core genome phylogeny, and a low recombination-to-mutation ratio. Unlike rapidly evolving genes that adapt to evade host immune responses, *ospA*’s specialized role in the tick vector (facilitating attachment to gut receptors essential for transmission) imposes unique evolutionary constraints^71^. These vector-specific pressures likely result in divergence from the core genome phylogeny despite minimal recombination, as observed in other vector-adaptive genes^72^.

In contrast, *ospB*, while also showing a low recombination-to-mutation ratio, exhibits moderate congruence with the core genome (i.e., reaching 75% of congruence reading the MLST classification method). *ospB* is expressed in both the tick and mammalian environments and plays a role in immune evasion, which exposes it to broader selective pressures across both ecological contexts^73^. These dual selective pressures likely drive *ospB*’s evolution in a way that partially aligns with the core genome, as it needs to retain functional integrity across diverse environments. P45-13, encoded by the *bba57* gene^74^, shows similar evolutionary patterns to *ospB* with a low recombination-to-mutation ratio (0.08) and moderate congruence with the core genome. Like *ospB*, P45-13 fulfills roles in both the tick and mammalian environments, acting as a surface-exposed lipoprotein critical for establishing infection^75^.

### Geographic structuring and population dynamics

*Borrelia burgdorferi* s.s. populations in Canada show strong geographic structuring. Nova Scotia strains were the most clonal (haplotype/singleton ratio = 4), reflecting limited recombination and gene flow, while Ontario/Manitoba strains were more diverse and interconnected. Modularity analysis confirmed compartmentalized clusters in NS versus higher connectivity in ON/MB, consistent with host/vector movement patterns^19,28^.

This pattern may be influenced by the movement of hosts or other environmental factors that facilitate greater genetic exchange between clusters in this region^18^. Seven of the 12 phylogenetic groups were recovered in network analysis, including distinct NS lineages such as Group 2 (B31-like) and Group 5 (close to 297). By contrast, ON/MB strains showed multiple interconnections, e.g., Group 1 linked to reference strains 156a (ON) and ZS7, 118a, B331 (MB). These results suggest stronger local adaptation in NS and higher genetic exchange in ON/MB^18,19,76^. This could suggest that these regions are geographically better connected, facilitating the movement of hosts and vectors (but see below), which would contribute to increased genetic exchange within *B. burgdorferi* s.s. populations^28,54,77,78^. The network analysis identified more interconnecting edges among groups in these regions, which suggests greater genetic exchange between strains and shared genomic components. For instance, Group 1 in Ontario aligns with the 156a strain, while in Manitoba, Group 1 shows genetic ties to ZS7, 118a, and B331 strains. This regional variation in genetic affinity is primarily observed in genes of the accessory genome, while the core genome remains consistent between Ontario and Manitoba, with the chromosome closely resembling ZS7. The connectivity between Groups 1 and 8 in MB, which share multiple markers like IGS-1 A and RSP2, indicates ongoing gene flow and shared evolutionary history, a phenomenon supported by earlier studies that reported high genetic exchange rates within *B. burgdorferi* s.s populations in interconnected landscapes^56,79^.

Further integrated field and laboratory studies are needed to explore exactly why these geographic differences exist. It is possible that the hills that run along the centre of NS limit connectivity amongst populations of ticks and *B. burgdorferi* s.s. that were likely founded by ticks introduced by migratory birds. Certainly, this could account for segregation of Pictou (which is on the north coast of NS) from the other sites where ticks were collected (which are on the south coast). However, there are no obvious geographical barriers (e.g. mountains or rivers) between the three sites on the south coast. Geographic separation of sites on the south coast of Nova Scotia (170 km from the Shelburne site to the Bedford site) is similar to that for the northeastern Ontario sites (approximately 110 km from Birch island to Manitou Rapids), but in the latter region, two of the sites are on islands in Lake of the Woods (Birch Island and Big Island) (see Fig. 1 in^35^. So connectivity amongst sites in both regions is most likely by migratory birds moving ticks and *B. burgdorferi* s.s. around. Perhaps another key factor distinguishing the ecology of *B. burgdorferi* s.s. in the two regions is the seasonal synchrony of activity of nymphal and larval *I. scapularis* in central regions of North America^80^. In northeastern North America, nymphal *I. scapularis* are mostly active in spring, while larvae are active in late summer, and this means northward migrating birds in spring carry almost exclusively nymphal *I. scapularis*, which moult into adult ticks that rarely feed on competent reservoir hosts. Consequently, although birds themselves can be infected and may occasionally transmit *B. burgdorferi* s.s. to *larvae*, dispersal driven primarily by spring-migrating birds (versus year-round dispersal by terrestrial hosts) creates a bottleneck to connectivity amongst *B. burgdorferi* s.s. populations in northeastern North America^54^. In contrast, in central regions spring migrating birds carry both nymphs and larvae, and larvae moult into nymphs that will feed on and (if infected) infect reservoir hosts. As a consequence introduction of *B. burgdorferi* s.s. into emerging *I. scapularis* populations appears to be much more efficient^54^, and we speculate that this permits greater connectivity of *B. burgdorferi* s.s. populations in this region.

### Core and accessory genome co-evolution

Core–accessory co-evolution was evident. Positive correlations between *vlsE1* (C6 peptide) and chromosomal genes (*bmpA*, *flaB*, MLST loci) suggest shared selective pressures linked to immune evasion and host colonization^81,82^. The IR6 region of *vlsE1* is highly conserved within *B. burgdorferi* s.s^83^, making it a reliable diagnostic target, though it varies markedly across other *Borrelia* genospecies^84^.

In contrast, *ospC* showed negative correlations with core genes such as *oms66* and P83-100, reflecting its high recombination and antigenic diversity, whereas these chromosomal genes remain conserved due to essential functional roles^10,84–87^.

### Implications for Lyme disease epidemiology and diagnostics

The higher clonality observed in NS indicates a more genetically homogeneous population of *B. burgdorferi* s.s., which may simplify predictive modeling of spread and persistence. While genetic diversity could theoretically influence antigenic variability and diagnostic sensitivity, our study did not directly assess diagnostic performance, and current evidence from North America does not support a need for region-specific diagnostics^87–89^.

Our own unpublished exploratory serological studies in mice likewise did not reveal convincing differences in diagnostic performance across strains, although infection dynamics did vary by genotype^89,90^.

Geographic clustering of specific genotypes, such as the RST1/RSP1/ST1 clade associated with disseminated disease in NS, highlights the value of genomic surveillance for identifying and tracking potentially high-risk clades. This emphasizes the importance of continued monitoring of population structure to support public health strategies for early detection and intervention.

## Conclusion

This study emphasizes the importance of considering both core and accessory genome components in understanding *B. burgdorferi* s.s. evolution. While the core genome provides a stable framework reflecting the evolutionary backbone of the bacterium, plasmid-encoded genes, particularly those involved in host-pathogen interactions, demonstrate substantial plasticity due to recombination events. This dynamic adaptability of the accessory genome enables *B. burgdorferi* s.s. to exploit a wide range of ecological niches and host environments, supporting its persistence across diverse geographic regions^8,19^.

Future studies should expand on these findings by increasing sample sizes and including broader geographic sampling across North America. Such efforts will help refine our understanding of the genetic landscape of *B. burgdorferi* s.s. and its evolutionary drivers, ultimately informing genomic surveillance and public health strategies for Lyme disease across different ecological regions.

## Supplementary Information

Below is the link to the electronic supplementary material.

Supplementary Material 1

Supplementary Material 2

Supplementary Material 3

Supplementary Material 4

Supplementary Material 5

Supplementary Material 6

Supplementary Material 7

Supplementary Material 8

Supplementary Material 9

Supplementary Material 10

Supplementary Material 11

Supplementary Material 12

Supplementary Material 13

Supplementary Material 14

## Data Availability

The datasets generated and/or analysed during the current study are available in the NCBI SRA under BioProject ID PRJNA416494 [https://www.ncbi.nlm.nih.gov/bioproject/PRJNA416494], and in the supplementary information files. All data generated or analysed during this study are included in this published article [and its supplementary information files].

## References

[1] Rudenko, N., Golovchenko, M., Grubhoffer, L. & Oliver, J. H. Updates on *Borrelia burgdorferi* sensu Lato complex with respect to public health. *Ticks Tick-Borne Dis.* **2**, 123–128 (2011). 21890064 10.1016/j.ttbdis.2011.04.002 PMC3167092

[2] Gern, L. *Borrelia burgdorferi* sensu lato, the agent of Lyme borreliosis: life in the wilds. *Parasite* **15**, 244–247 (2008). 18814688 10.1051/parasite/2008153244

[3] Kahl, O., Gern, L., Eisen, L. & Lane, R. S. Ecological research on *Borrelia burgdorferi* sensu lato: terminology and some methodological pitfalls. *Lyme Borreliosis: Biology Epidemiol. Control*. 29–46. 10.1079/9780851996325.0029 (2002).

[4] Wolcott, K. A., Margos, G., Fingerle, V. & Becker, N. S. Host association of *Borrelia burgdorferi* sensu lato: A review. *Ticks Tick-Borne Dis.* **12**, 101766 (2021). 34161868 10.1016/j.ttbdis.2021.101766

[5] Margos, G. et al. MLST of housekeeping genes captures geographic population structure and suggests a European origin of borrelia burgdorferi. *Proc. Natl. Acad. Sci. U S A*. **105**, 8730–8735 (2008). 18574151 10.1073/pnas.0800323105 PMC2435589

[6] Margos, G. et al. PubMLST.org – The new home for the borrelia MLSA database. *Ticks Tick-Borne Dis.* **6**, 869–871 (2015). 26115778 10.1016/j.ttbdis.2015.06.007

[7] Bunikis, J. et al. Sequence typing reveals extensive strain diversity of the Lyme borreliosis agents *Borrelia burgdorferi* in North America and borrelia afzelii in Europe. *Microbiology* **150**, 1741–1755 (2004). 15184561 10.1099/mic.0.26944-0

[8] Fraser, C. M. et al. Genomic sequence of a Lyme disease spirochaete, *Borrelia burgdorferi*. *Nature* **390**, 580–586 (1997). 9403685 10.1038/37551

[9] Wang, I. N. et al. Genetic diversity of OspC in a local population of *Borrelia burgdorferi* sensu stricto. *Genetics* **151**, 15–30 (1999). 9872945 10.1093/genetics/151.1.15 PMC1460459

[10] Barbour, A. G. & Travinsky, B. Evolution and distribution of the ospC gene, a transferable serotype determinant of *Borrelia burgdorferi*. *mBio* **1**, e00153-10 (2010). 10.1128/mBio.00153-10 PMC2945197 20877579

[11] Brisson, D. & Dykhuizen, D. E. OspC diversity in *Borrelia burgdorferi*. *Genetics* **168**, 713–722 (2004). 15514047 10.1534/genetics.104.028738 PMC1448846

[12] Rudenko, N. et al. Detection of *Borrelia burgdorferi* sensu stricto *OspC* alleles associated with human Lyme borreliosis worldwide in Non-Human-Biting tick Ixodes affinis and rodent hosts in southeastern united States. *Appl. Environ. Microbiol.* **79**, 1444–1453 (2013). 23263953 10.1128/AEM.02749-12 PMC3591949

[13] Travinsky, B., Bunikis, J. & Barbour, A. G. Geographic differences in genetic locus linkages for *Borrelia burgdorferi*. *Emerg. Infect. Dis.* **16**, 1147–1150 (2010). 20587192 10.3201/eid1607.091452 PMC3321895

[14] Hanincova, K. et al. Multilocus sequence typing of *Borrelia burgdorferi* suggests existence of lineages with differential pathogenic properties in humans. *PLoS ONE*. **8**, e73066 (2013). 24069170 10.1371/journal.pone.0073066 PMC3775742

[15] Liveris, D. et al. Genetic diversity of *Borrelia burgdorferi* in Lyme disease patients as determined by culture versus direct PCR with clinical specimens. *J. Clin. Microbiol.* **37**, 565–569 (1999). 9986813 10.1128/jcm.37.3.565-569.1999 PMC84470

[16] Seinost, G. et al. Four clones of *Borrelia burgdorferi* sensu stricto cause invasive infection in humans. *Infect. Immun.* **67**, 3518–3524 (1999). 10377134 10.1128/iai.67.7.3518-3524.1999 PMC116539

[17] Lemieux, J. E. et al. Whole genome sequencing of human *Borrelia burgdorferi* isolates reveals linked blocks of accessory genome elements located on plasmids and associated with human dissemination. *PLOS Pathog*. **19**, e1011243 (2023). 37651316 10.1371/journal.ppat.1011243 PMC10470944

[18] Mechai, S. et al. Evidence for Host-Genotype associations of *Borrelia burgdorferi* sensu stricto. *PloS One*. **11**, e0149345 (2016). 26901761 10.1371/journal.pone.0149345 PMC4763156

[19] Margos, G. et al. Two boundaries separate *Borrelia burgdorferi* populations in North America. *Appl. Environ. Microbiol.* **78**, 6059–6067 (2012). 22729536 10.1128/AEM.00231-12 PMC3416618

[20] Hoen, A. G. et al. Phylogeography of *Borrelia burgdorferi* in the eastern United States reflects multiple independent Lyme disease emergence events. *Proc. Natl. Acad. Sci.* **106**, 15013–15018 (2009). 10.1073/pnas.0903810106 PMC2727481 19706476

[21] Spielman, A. The emergence of Lyme disease and human babesiosis in a changing environmenta. *Ann. N Y Acad. Sci.* **740**, 146–156 (1994). 7840446 10.1111/j.1749-6632.1994.tb19865.x

[22] Ginsberg, H. S. et al. Why Lyme disease is common in the Northern US, but rare in the south: the roles of host choice, host-seeking behavior, and tick density. *PLOS Biol.* **19**, e3001066 (2021). 33507921 10.1371/journal.pbio.3001066 PMC7842935

[23] Madhav, N. K., Brownstein, J. S., Tsao, J. I. & Fish, D. A dispersal model for the range expansion of blacklegged tick (Acari: Ixodidae). *J. Med. Entomol.* **41**, 842–852 (2004). 15535611 10.1603/0022-2585-41.5.842

[24] Kilpatrick, A. M. et al. Lyme disease ecology in a changing world: consensus, uncertainty and critical gaps for improving control. *Philos. Trans. R Soc. B Biol. Sci.* **372**, 20160117 (2017). 10.1098/rstb.2016.0117 PMC5413869 28438910

[25] Ebi, K. L., Ogden, N. H., Semenza, J. C. & Woodward, A. Detecting and attributing health burdens to climate change. *Environ. Health Perspect.* **125**, 085004 (2017). 28796635 10.1289/EHP1509 PMC5783629

[26] McPherson, M. et al. Expansion of the Lyme disease vector *Ixodes scapularis* in Canada inferred from CMIP5 climate projections. *Environ. Health Perspect.* **125**, 057008 (2017). 28599266 10.1289/EHP57 PMC5730520

[27] Ogden, N. H. Vector-borne disease, climate change and urban design. *Can. Commun. Dis. Rep. Releve Mal Transm Au Can.* **42**, 202 (2016). 10.14745/ccdr.v42i10a04 PMC5757744 29769980

[28] Ogden, N. H. et al. Role of migratory birds in introduction and range expansion of *Ixodes scapularis* ticks and of *Borrelia burgdorferi* and *Anaplasma phagocytophilum* in Canada. *Appl. Environ. Microbiol.* **74**, 1780–1790 (2008). 18245258 10.1128/AEM.01982-07 PMC2268299

[29] Mechai, S., Margos, G., Feil, E. J., Lindsay, L. R. & Ogden, N. H. Complex population structure of *Borrelia burgdorferi* in southeastern and South central Canada as revealed by phylogeographic analysis. *Appl. Environ. Microbiol.* **81**, 1309–1318 (2015). 25501480 10.1128/AEM.03730-14 PMC4309700

[30] Kurtenbach, K. et al. Fundamental processes in the evolutionary ecology of Lyme borreliosis. *Nat. Rev. Microbiol.* **4**, 660–669 (2006). 16894341 10.1038/nrmicro1475

[31] Ogden et al. Evolutionary aspects of emerging Lyme disease in Canada. *Appl. Environ. Microbiol.* **81**, 7350–7359 (2015). 26296723 10.1128/AEM.01671-15 PMC4592865

[32] Jacquot, M. et al. High-Throughput sequence typing reveals genetic differentiation and host specialization among populations of the *Borrelia burgdorferi* species complex that infect rodents. *PLoS ONE*. **9**, e88581 (2014). 24533116 10.1371/journal.pone.0088581 PMC3922933

[33] Jacquot, M. et al. Comparative population genomics of the *Borrelia burgdorferi* species complex reveals high degree of genetic isolation among species and underscores benefits and constraints to studying Intra-Specific epidemiological processes. *PLoS ONE*. **9**, e94384 (2014). 24721934 10.1371/journal.pone.0094384 PMC3993988

[34] Seifert, S. N., Khatchikian, C. E., Zhou, W. & Brisson, D. Evolution and population genomics of the Lyme borreliosis pathogen, *Borrelia burgdorferi*. *Trends Genet.* **31**, 201–207 (2015). 25765920 10.1016/j.tig.2015.02.006 PMC4380588

[35] Tyler, S. et al. Whole genome sequencing and phylogenetic analysis of strains of the agent of Lyme disease *Borrelia burgdorferi* from Canadian emergence zones. *Sci. Rep.* **8**, 10552 (2018). 30002414 10.1038/s41598-018-28908-7 PMC6043495

[36] Minh, B. Q. et al. IQ-TREE 2: new models and efficient methods for phylogenetic inference in the genomic era. *Mol. Biol. Evol.* **37**, 1530–1534 (2020). 32011700 10.1093/molbev/msaa015 PMC7182206

[37] Tamura, K. et al. MEGA5: molecular evolutionary genetics analysis using maximum Likelihood, evolutionary Distance, and maximum parsimony methods. *Mol. Biol. Evol.* **28**, 2731–2739 (2011). 21546353 10.1093/molbev/msr121 PMC3203626

[38] Katoh, K., Misawa, K., Kuma, K. & Miyata, T. MAFFT: a novel method for rapid multiple sequence alignment based on fast fourier transform. *Nucleic Acids Res.* **30**, 3059–3066 (2002). 12136088 10.1093/nar/gkf436 PMC135756

[39] Katoh, K. & Standley, D. M. MAFFT multiple sequence alignment software version 7: improvements in performance and usability. *Mol. Biol. Evol.* **30**, 772–780 (2013). 23329690 10.1093/molbev/mst010 PMC3603318

[40] De Vienne, D. M., Giraud, T. & Martin, O. C. A congruence index for testing topological similarity between trees. *Bioinformatics* **23**, 3119–3124 (2007). 17933852 10.1093/bioinformatics/btm500

[41] Leigh, J. W., Lapointe, F. J., Lopez, P. & Bapteste, E. Evaluating phylogenetic congruence in the Post-Genomic era. *Genome Biol. Evol.* **3**, 571–587 (2011). 21712432 10.1093/gbe/evr050 PMC3156567

[42] Planet, P. J. Tree disagreement: measuring and testing incongruence in phylogenies. *J. Biomed. Inf.* **39**, 86–102 (2006). 10.1016/j.jbi.2005.08.008 16243006

[43] Didelot, X., Wilson, D. J. & ClonalFrameML Efficient inference of recombination in whole bacterial genomes. *PLOS Comput. Biol.* **11**, e1004041 (2015). 25675341 10.1371/journal.pcbi.1004041 PMC4326465

[44] McNally, A. et al. Combined analysis of variation in Core, accessory and regulatory genome regions provides a Super-Resolution view into the evolution of bacterial populations. *PLOS Genet.* **12**, e1006280 (2016). 27618184 10.1371/journal.pgen.1006280 PMC5019451

[45] Touchon, M. et al. Phylogenetic background and habitat drive the genetic diversification of Escherichia coli. *PLOS Genet.* **16**, e1008866 (2020). 32530914 10.1371/journal.pgen.1008866 PMC7314097

[46] DATAtab Team. DATAtab: online statistics calculator. DATAtab eU Graz, Austria. *URL Httpsdatatab Net*. (2024).

[47] Nascimento, M. et al. PHYLOViZ 2.0: providing scalable data integration and visualization for multiple phylogenetic inference methods. *Bioinformatics* **33**, 128–129 (2017). 27605102 10.1093/bioinformatics/btw582

[48] Mann, H. B. & Whitney, D. R. On a test of whether one of two random variables is stochastically larger than the other. *Ann. Math. Stat.* **18**, 50–60 (1947).

[49] Nachar, N. & The Mann-Whitney, U. A test for assessing whether two independent samples come from the same distribution. *Tutor. Quant. Methods Psychol.* **4**, 13–20 (2008).

[50] Cliff, N. Dominance statistics: ordinal analyses to answer ordinal questions. *Psychol. Bull.* **114**, 494–509 (1993).

[51] Virtanen, P. et al. SciPy 1.0: fundamental algorithms for scientific computing in python. *Nat. Methods*. **17**, 261–272 (2020). 32015543 10.1038/s41592-019-0686-2 PMC7056644

[52] Ben-Shachar, M., Lüdecke, D. & Makowski, D. Estimation of effect size indices and standardized parameters. *J. Open. Source Softw.* **5**, 2815 (2020).

[53] Bastian, M., Heymann, S. & Jacomy, M. Gephi: an open source software for exploring and manipulating networks. in *Proceedings of the international AAAI conference on web and social media* **3**, 361–362 (2009).

[54] Ogden, N. H., Mechai, S. & Margos, G. Changing geographic ranges of ticks and tick-borne pathogens: drivers, mechanisms and consequences for pathogen diversity. *Front. Cell. Infect. Microbiol.* **3**, 46 (2013). 24010124 10.3389/fcimb.2013.00046 PMC3756306

[55] Hanincová, K. et al. Fitness variation of *Borrelia burgdorferi* sensu stricto strains in mice. *Appl. Environ. Microbiol.* **74**, 153–157 (2008). 17981941 10.1128/AEM.01567-07 PMC2223198

[56] Walter, K. S., Carpi, G., Caccone, A. & Diuk-Wasser, M. A. Genomic insights into the ancient spread of Lyme disease across North America. *Nat. Ecol. Evol.* **1**, 1569–1576 (2017). 29185509 10.1038/s41559-017-0282-8 PMC6431794

[57] Brisson, D., Drecktrah, D., Eggers, C. H. & Samuels, D. S. Genetics of *Borrelia burgdorferi*. *Annu. Rev. Genet.* **46**, 515–536 (2012). 22974303 10.1146/annurev-genet-011112-112140 PMC3856702

[58] Margos, G., Fingerle, V. & Reynolds, S. Borrelia bavariensis: vector Switch, niche Invasion, and geographical spread of a Tick-Borne bacterial parasite. *Front. Ecol. Evol.* **7**, 401 (2019).

[59] Akther, S. et al. Natural selection and recombination at host-interacting lipoprotein loci drive genome diversification of Lyme disease and related bacteria. *mBio* **15**, e01749–e01724 (2024). 39145656 10.1128/mbio.01749-24 PMC11389397

[60] Dykhuizen, D. E. et al. The propensity of different *Borrelia burgdorferi* sensu stricto genotypes to cause disseminated infections in humans. *Am. J. Trop. Med. Hyg.* **78**, 806–810 (2008). 18458317 PMC2387051

[61] Ogden, N. et al. Investigation of genotypes of *Borrelia burgdorferi* in *Ixodes scapularis* ticks collected during surveillance in Canada. *Appl. Environ. Microbiol.* **77**, 3244–3254 (2011). 21421790 10.1128/AEM.02636-10 PMC3126474

[62] Russell, J. N. et al. Whole-genome sequencing of Western Canadian borrelia spp. Collected from diverse tick and animal hosts reveals short-lived local genotypes interspersed with longer-lived continental genotypes. *Microb Genomics* **10**, 001276 (2024). 10.1099/mgen.0.001276 PMC11296321 39093316

[63] Bedford, N. L. & Hoekstra, H. E. Peromyscus mice as a model for studying natural variation. *eLife* **4**, e06813 (2015). 26083802 10.7554/eLife.06813 PMC4470249

[64] Jahan, N. A., Lindsey, L. L. & Larsen, P. A. The role of peridomestic rodents as reservoirs for zoonotic foodborne pathogens. *Vector-Borne Zoonotic Dis.* **21**, 133–148 (2021). 33351736 10.1089/vbz.2020.2640

[65] Vuong, H. B. et al. Occurrence and transmission efficiencies of *Borrelia burgdorferi* OspC types in avian and mammalian wildlife. *Infect. Genet. Evol. J. Mol. Epidemiol. Evol. Genet. Infect. Dis.* **27**, 594–600 (2014). 10.1016/j.meegid.2013.12.011 PMC4180015 24382473

[66] Castillo-Ramírez, S. et al. Trans-Atlantic exchanges have shaped the population structure of the Lyme disease agent *Borrelia burgdorferi* sensu stricto. *Sci. Rep.* **6**, 22794 (2016). 26955886 10.1038/srep22794 PMC4783777

[67] Didelot, X. & Maiden, M. C. J. Impact of recombination on bacterial evolution. *Trends Microbiol.* **18**, 315–322 (2010). 20452218 10.1016/j.tim.2010.04.002 PMC3985120

[68] Johansson, A. et al. Worldwide genetic relationships among *Francisella tularensis* isolates determined by Multiple-Locus Variable-Number tandem repeat analysis. *J. Bacteriol.* **186**, 5808–5818 (2004). 15317786 10.1128/JB.186.17.5808-5818.2004 PMC516809

[69] Morelli, G. et al. Yersinia pestis genome sequencing identifies patterns of global phylogenetic diversity. *Nat. Genet.* **42**, 1140–1143 (2010). 21037571 10.1038/ng.705 PMC2999892

[70] Grimm, D. et al. Outer-surface protein C of the Lyme disease spirochete: A protein induced in ticks for infection of mammals. *Proc. Natl. Acad. Sci.* **101**, 3142–3147 (2004). 14970347 10.1073/pnas.0306845101 PMC365757

[71] Pal, U. et al. TROSPA, an *Ixodes scapularis* receptor for *Borrelia burgdorferi*. *Cell* **119**, 457–468 (2004). 15537536 10.1016/j.cell.2004.10.027

[72] De Silva, A. M., Telford, S. R., Brunet, L. R., Barthold, S. W. & Fikrig, E. *Borrelia burgdorferi* OspA is an arthropod-specific transmission-blocking Lyme disease vaccine. *J. Exp. Med.* **183**, 271–275 (1996). 8551231 10.1084/jem.183.1.271 PMC2192397

[73] Neelakanta, G. et al. Outer surface protein B is critical for *Borrelia burgdorferi* adherence and survival within Ixodes ticks. *PLoS Pathog*. **3**, e33 (2007). 17352535 10.1371/journal.ppat.0030033 PMC1817655

[74] Schwartz, I., Margos, G., Casjens, S. R., Qiu, W. G. & Eggers, C. H. Multipartite genome of Lyme disease *Borrelia*: Structure, variation and prophages. *Curr. Issues Mol. Biol.* 409–454. 10.21775/cimb.042.409 (2022). 10.21775/cimb.042.409 33328355

[75] Yang, X. et al. Novel microbial virulence factor triggers murine Lyme arthritis. *J. Infect. Dis.* **207**, 907–918 (2013). 23303811 10.1093/infdis/jis930 PMC3571445

[76] Mechai, S. al, et. The population structure of *Borrelia burgdorferi* in Canada. *PLoS One*. **10**, e0141775 (2015).26509445

[77] Morshed, M. G. et al. Migratory songbirds disperse ticks across Canada, and first isolation of the Lyme disease spirochete, *Borrelia burgdorferi*, from the avian tick, Ixodes auritulus. *J. Parasitol.* **91**, 780–790 (2005). 17089744 10.1645/GE-3437.1

[78] Ogden, N. H. et al. Investigation of relationships between temperature and developmental rates of tick *Ixodes scapularis* (Acari: Ixodidae) in the laboratory and field. *J. Med. Entomol.* **41**, 622–633 (2004). 15311453 10.1603/0022-2585-41.4.622

[79] Dykhuizen, D. E. & Baranton, G. *Borrelia burgdorferi*: a (somewhat) clonal bacterial species. *Trends Microbiol.* **9**, 472 (2001). 11597442 10.1016/s0966-842x(01)02139-4

[80] Gatewood, A. G. et al. Climate and tick seasonality are predictors of *Borrelia burgdorferi* genotype distribution. *Appl. Environ. Microbiol.* **75**, 2476–2483 (2009). 19251900 10.1128/AEM.02633-08 PMC2675205

[81] Boyle, W. K. et al. DksA-dependent regulation of RpoS contributes to *Borrelia burgdorferi* tick-borne transmission and mammalian infectivity. *PLOS Pathog*. **17**, e1009072 (2021). 33600418 10.1371/journal.ppat.1009072 PMC7924775

[82] Caimano, M. J. et al. The RpoS gatekeeper in *Borrelia burgdorferi*: an invariant regulatory scheme that promotes spirochete persistence in reservoir hosts and niche diversity. *Front. Microbiol.* **10**, 1923 (2019). 31507550 10.3389/fmicb.2019.01923 PMC6719511

[83] Liang, F. T., Nowling, J. M. & Philipp, M. T. Cryptic and exposed invariable regions of VlsE, the variable surface antigen of *Borrelia burgdorferi* Sl. *J. Bacteriol.* **182**, 3597–3601 (2000). 10852896 10.1128/jb.182.12.3597-3601.2000 PMC101975

[84] Sillanpaa, H. et al. Immune responses to borrelial VlsE IR6 peptide variants. *Int. J. Med. Microbiol.* **297**, 45–52 (2007). 17234451 10.1016/j.ijmm.2006.09.001

[85] Coburn, J. & Cugini, C. Targeted mutation of the outer membrane protein P66 disrupts attachment of the Lyme disease agent, *Borrelia burgdorferi*, to integrin α_v_ β_3_. *Proc. Natl. Acad. Sci.* **100**, 7301–7306 (2003). 10.1073/pnas.1131117100 PMC165870 12748384

[86] Skare, J. T. et al. Porin activity of the native and Recombinant outer membrane protein Oms28 of *Borrelia burgdorferi*. *J. Bacteriol.* **178**, 4909–4918 (1996). 8759855 10.1128/jb.178.16.4909-4918.1996 PMC178274

[87] Waddell, L. A. et al. The accuracy of diagnostic tests for Lyme disease in Humans, A systematic review and Meta-Analysis of North American research. *PLOS ONE*. **11**, e0168613 (2016). 28002488 10.1371/journal.pone.0168613 PMC5176185

[88] Gutiérrez, J., Rodriguez, M. A. & Maroto, M. C. Applications of polymerase chain reaction to diagnose Lyme borreliosis. *Serodiagn Immunother Infect. Dis.* **7**, 109–113 (1995).

[89] Zinck, C. B. et al. *Borrelia burgdorferi* strain and host sex influence pathogen prevalence and abundance in the tissues of a laboratory rodent host. *Mol. Ecol.* **31**, 5872–5888 (2022). 36112076 10.1111/mec.16694 PMC12359074

[90] Zinck, C. B. et al. Variation among strains of *Borrelia burgdorferi* in host tissue abundance and lifetime transmission determine the population strain structure in nature. *PLoS Pathog*. **19**, e1011572 (2023). 37607182 10.1371/journal.ppat.1011572 PMC10473547

