# Supplementary material for: Genetic landscape of Borrelia burgdorferi sensu stricto in Canada: a study of genetic diversity
Source: Sci Rep. 2025 Nov 20;15:40954. doi: 10.1038/s41598-025-24758-2 (PMC12635250; doi:10.1038/s41598-025-24758-2)
Supplement: Supplementary file 1 — Supplementary Material 1 [file 41598_2025_24758_MOESM1_ESM.docx]

Supplementary information for: Samir Mechai, Edward J. Feil, Gabrielle Margos, Nick H. Ogden. Genetic landscape of *Borrelia burgdorferi* sensu stricto in Canada: a study of genetic diversity.

Table S1: Characteristics and functional annotations of 24 *Borrelia burgdorferi* sensu stricto gene products and loci analyzed in this study. This table summarizes both plasmid-encoded and chromosomal markers included in the analysis. Genes comprise 11 plasmid-encoded surface-exposed proteins (C6 peptide of the *VlsE1* gene, *dbpA*, *dbpB*, fibronectin-binding protein P35, *oms28*, *ospA*, *ospB*, *ospC*, *ospD*, P37, P45-13), four chromosomal antigens (*BmpA*, *FlaB*, *oms66*, P83-100), eight housekeeping genes from the MLST scheme (*clpA*, *clpX*, *nifS*, *pepX*, *pyrG*, *recG*, *rplB*, *uvrA*), and three ribosomal markers (16S–23S intergenic spacer [IGS], restriction site types [RST], ribosomal spacer pattern [RSP]). Functional roles in infection, host–pathogen interactions, immune evasion, and phylogenetic classification are indicated.

| Locus | Function | References | Plasmid Location | GenBank Accession (B31) |
| --- | --- | --- | --- | --- |
| *VlsE1* (C6) | Immune evasion via antigenic variation; key in host-pathogen interactions | ^1^ | lp28-1 | U76405.1 |
| *dbpA* | Binds decorin for adherence to host connective tissues | ^2–4^ | lp54 | BB_A24 |
| *dbpB* | Binds decorin for adherence to host connective tissues | ^2–4^ | lp54 | BB_A25 |
| Fibronectin P35 | Facilitates adhesion to fibronectin, aiding early tissue colonization | ^5^ | lp36 | BB_K32 |
| *oms28* | Surface lipoprotein involved in immune evasion and persistence | ^6,7^ | lp54 | BB_A74 |
| *ospA* | Tick midgut binding during tick-to-mammal transmission | ^8,9^ | lp54 | BB_A15 |
| *ospB* | Aids immune evasion during tick-to-mammal transmission | ^10^ | lp54 | BB_A16 |
| *ospC* | Critical for tick salivary gland and/or early mammalian infection; aids immune evasion and infection establishment | ^11,12^ | cp26 | BB_B19 |
| *ospD* | Facilitates infection establishment | ^13^ | lp38 | BB_J09 |
| P37 | Surface-exposed lipoprotein likely aiding tissue adherence | ^14^ | lp36 | BB_K50 |
| P45-13 | Modulates host immune responses for early pathogen survival | ^15^ | lp54 | BB_A57 |
| *BmpA* | Antigen involved in immune evasion | ^16^ | Chromosome | BB_0383 |
| *FlaB* | Plays a role in motility and immune evasion | ^12^ | Chromosome | BB_0147 |
| *oms66* | Integrin-binding protein; aids in host cell adhesion | ^16^ | Chromosome | BB_0603 |
| P83-100 | Protoplasmic cylinder-associated protein | ^17^ | Chromosome | BB_0744 |
| *clpA* | Critical for protein degradation and quality control | ^18^ | Chromosome | BB_0369 |
| *clpX* | Works with ClpP for protein turnover | ^18^ | Chromosome | BB_0612 |
| *nifS* | Involved in cofactor biosynthesis | ^18^ | Chromosome | BB_0084 |
| *pepX* | Role in peptide metabolism | ^18^ | Chromosome | BB_0627 |
| *pyrG* | Synthesizes CTP | ^18^ | Chromosome | BB_0575 |
| *recG* | Involved in DNA repair | ^18^ | Chromosome | BB_0581 |
| *rplB* | Integral to protein synthesis | ^18^ | Chromosome | BB_0481 |
| *uvrA* | DNA repair | ^18^ | Chromosome | BB_0837 |
| *rrs*-*rrlA* | Noncoding 16S–23S intergenic spacer | ^19^ | Chromosome | NC_001318.1 |

Table S2: Genomic data sources and characteristics of 24 reference strains of *Borrelia burgdorferi* sensu stricto used to characterize the 64 Canadian genomes analyzed in this study. The table lists each reference strain along with its geographic origin, host of isolation, assembly submitter, and corresponding BioProject accession number. These reference strains provided a comparative framework for phylogenetic analyses, population structure evaluation, and network-based modularity assessment.

| Strain | Geographic Location | Host | Assembly Submitter | BioProject Accession |
| --- | --- | --- | --- | --- |
| 118a | New York, USA | Human | J. Craig Venter Institute | PRJNA21001 |
| 156a | New York, USA | Human | J. Craig Venter Institute | PRJNA19835 |
| 297 | New York, USA | Human | IBIS, Laval University | PRJNA1130942 |
| 29805 | Connecticut, USA | Tick | J. Craig Venter Institute | PRJNA28621 |
| 64b | New York, USA | Human | J. Craig Venter Institute | PRJNA28633 |
| 72a | New York, USA | Human | J. Craig Venter Institute | PRJNA21003 |
| 94a | New York, USA | Human | J. Craig Venter Institute | PRJNA20999 |
| B31 | Shelter Island, New York, USA | Tick | IBIS, Laval University | PRJNA1130942 |
| B31_NRZ | Germany | Tick | Bavarian Health and Food Safety Authority | PRJNA270494 |
| B31-5A4 | New York, USA | Tick | Columbia University | PRJNA820963 |
| B331 | New York, USA | Human | New York Medical College | PRJNA342676 |
| B356 | New York, USA | Human | Columbia University | PRJNA820963 |
| B379 | New York, USA | Human | Columbia University | PRJNA820963 |
| B408 | New York, USA | Human | Columbia University | PRJNA820963 |
| B500 | Westchester, New York, USA | Human | New York Medical College | PRJNA342676 |
| Bol26 | Italy | Human | J. Craig Venter Institute | PRJNA19837 |
| CA-11.2A | California, USA | Tick | J. Craig Venter Institute | PRJNA28629 |
| JD1 | Massachusetts, USA | Human | University of Maryland School of Medicine - IGS | PRJNA29359 |
| MM1 | Minnesota, USA | Mouse | Institute for Systems Biology | PRJNA397229 |
| N40 | New York, USA | Tick | University of Maryland School of Medicine - IGS | PRJNA29357 |
| PAbe | Germany | Tick | Bavarian Health and Food Safety Authority | PRJNA270494 |
| Pali | Germany | Tick | Bavarian Health and Food Safety Authority | PRJNA270494 |
| ZS7 | Germany | Tick | J. Craig Venter Institute | PRJNA19839 |
| WI91-23 | Wisconsin, USA | Bird | J. Craig Venter Institute | PRJNA28627 |

Table S3: Comparative phylogenetic analysis of partitioned and concatenated evolutionary models for Borrelia burgdorferi sensu stricto genomes. Results from IQ-TREE (partitioned GTR per gene and concatenated GTR single-rate models) and MEGA (Jukes–Cantor model) are shown, including RF distances, log-likelihood values, and support from AU, SH, and KH tests.

| Feature | IQ-TREE (Partitioned GTR Model per gene) | IQ-TREE (Concatenated GTR Model, single rate) | MEGA (default settings) |
| --- | --- | --- | --- |
| Evolutionary model | GTR per gene (Partitioned) | GTR (Single model for all genes) | Jukes-Cantor (JC) model (equal substitution rates) |
| RF distance vs core genome | 44 | 36 | N/A |
| Normalized RF distance vs Core genome | 0.3385 | 0.2769 | N/A |
| RF distance (partition vs concatenated) | 10 | 10 | N/A |
| Normalized RF distance (partition vs concatenated) | 0.0769 | 0.0769 | N/A |
| logL (log-likelihood) | -22557.785 | -22557.732 | N/A |
| deltaL | 0.052 | 0.052 | N/A |
| p-AU (Approx. Unbiased Test) | 0.0235 | 0.976 | N/A |
| p-SH (Shimodaira-Hasegawa Test) | 0.0231 | 1 | N/A |
| p-KH (Kishino-Hasegawa Test) | 0.0259 | 0.974 | N/A |
| Model support & interpretation | Partition model rejected (p-AU < 0.05) | Concatenated model strongly supported | Core genome baseline reference |

Table S4: Geographic distribution of *Borrelia burgdorferi* sensu stricto phylogenetic groups. Distribution of 12 phylogenetic groups based on sequence types (STs) from the PubMLST database, with regional representation across the USA and Canada.

| **Phylogenetic Group** | **Sequence Types (ST)** | **Geographic distribution** |
| --- | --- | --- |
| Group 1 | ST55 | Midwestern USA (MN, WI), Mid-southwestern Canada (MB, ON) |
| Group 2 | ST1 | Northeastern USA (CT, MA, ME, NH, NY, PA, RI, VA, VT), Centrals-southeastern Canada (ON, QC, Maritimes) |
| Group 3 | ST46 | Midwestern USA (WI, MN), Mid-southwestern Canada (ON, MB) |
| Group 4 | ST51-ST268 | Northeastern USA (MN, WI, NY), Southwestern Canada (MB, BC) |
| Group 5 | ST3 | Northeastern USA Northeastern USA (MA, NY, CT, VT, VA, RI, NJ), Southeastern Canada (Maritimes, ON, QC) |
| Group 6 | ST4-ST32-ST740 | Northeastern and Midwest USA (CT, IL, MA, ME, MI, MN, NJ, NY, PA, RI, WI), South Canada (MB, ON, QC, Maritimes) |
| Group 7 | ST29 | Northeastern and Midwest USA (CT, IL, MN, WI), South Canada (BC, MB, ON, QC) |
| Group 8 | ST530 | Midwestern USA (WI, MN), Mid-southwestern Canada (MB, , NWON) |
| Group 9 | ST16-ST227-ST237-ST741 | Northeastern and Midwest USA (CT, MA, NY, RI, US, WI), South Canada (BC, MB, ON, QC, Maritimes) |
| Group 10 | ST12-ST221 | Midwest USA (CT, MA, NY, RI, WI, MI, IL), South Canada (BC, MB, ON, QC, Maritimes) |
| Group 11 | ST43 | Midwestern USA (WI, MN), South Midwestern Canada (BC, MB, NWON) |
| Group 12 | ST19-ST31-ST229 | Northeastern and Midwest USA (CT, NY, US Midwest), South Canada (MB, ON, QC, Maritimes) |

Table S5: Congruence between core genome and plasmid gene phylogenies using the MLST classification method. Congruence was scored based on monophyly and haplotype correspondence across 12 phylogenetic groups. “ab.” indicates the absence of the gene sequence for the corresponding taxon.

| Plasmid genes | Monophyletic groups (Core genome) | Group1 | Group2 | Group3 | Group4 | Group5 | Group6 | Group7 | Group8 | Group9 | Group10 | Group11 | Group12 | Congruence score |
| --- | --- | --- | --- | --- | --- | --- | --- | --- | --- | --- | --- | --- | --- | --- |
| *dpbA* | | 1 | 1 | 1 | 0 | 0 | 1 | 1 | 1 | 1 | 1 | 1 | 0 | 75% |
| *dpbB* | | 1 | 1 | 1 | 0 | 0 | 1 | 1 | 1 | 0 | 1 | 1 | 1 | 75% |
| Fibronectin P35 | | 1 | 1 | 1 | 1 | 1 | 1 | 1 | 1 | 1 | ab. | 1 | 0 | 91% |
| *oms28* | | 0 | 1 | 1 | 1 | 0 | 0 | 0 | 1 | 0 | 0 | 0 | 1 | 42% |
| *ospA* | | 1 | 1 | 1 | 0 | 0 | 0 | 0 | ab. | 0 | 0 | 0 | 0 | 27% |
| *ospB* | | 1 | 1 | 1 | 1 | 1 | 1 | 0 | 1 | 0 | 1 | 0 | 1 | 75% |
| *ospC* | | 1 | 1 | 1 | 1 | 1 | 1 | 0 | 1 | 0 | 0 | 1 | 1 | 75% |
| *ospD* | | 1 | 1 | ab. | 1 | ab. | ab. | ab. | 1 | 0 | 1 | 0 | 1 | 75% |
| P37 | | 1 | 1 | ab. | ab. | ab. | ab. | ab. | 1 | ab. | ab. | 1 | 1 | 100% |
| C6 | | 1 | 1 | 1 | 1 | 1 | ab. | 1 | ab. | 1 | 1 | 0 | ab. | 89% |
| P45-13 | | 1 | 1 | 1 | 0 | 1 | 1 | 0 | 1 | 0 | 1 | 0 | ab. | 64% |
| Congruence score | | 91% | 100% | 100% | 60% | 56% | 75% | 44% | 100% | 30% | 67% | 45% | 67% |  |

Table S6: Congruence between core genome and plasmid gene phylogenies using the *ospC* major group (MG) classification method. Congruence was evaluated for 12 phylogenetic groups based on monophyly and haplotype correspondence. “ab.” indicates the absence of the gene sequence for the corresponding taxon.

| Plasmid genes | Monophyletic groups (Core genome) | Group1 | Group2 | Group3 | Group4 | Group5 | Group6 | Group7 | Group8 | Group9 | Group10 | Group11 | Group12 | Congruence score |
| --- | --- | --- | --- | --- | --- | --- | --- | --- | --- | --- | --- | --- | --- | --- |
| *dpbA* | | 1 | 1 | 1 | 0 | 0 | 1 | 1 | 1 | 0 | 1 | 1 | 0 | 67% |
| *dpbB* | | 0 | 1 | 1 | 0 | 0 | 1 | 1 | 1 | 0 | 1 | 1 | 1 | 67% |
| Fibronectin P35 | | 0 | 0 | 0 | 1 | 1 | 1 | 1 | 1 | 1 | ab. | 1 | 0 | 64% |
| *oms28* | | 0 | 0 | 1 | 1 | 0 | 0 | 1 | 1 | 0 | 0 | 0 | 1 | 42% |
| *ospA* | | 0 | 1 | 1 | 0 | 0 | 0 | 0 | ab. | 0 | 0 | 0 | 1 | 27% |
| *ospB* | | 1 | 1 | 0 | 1 | 1 | 1 | 0 | 1 | 0 | 1 | 0 | 1 | 67% |
| *ospC* | | 1 | 1 | 1 | 1 | 1 | 1 | 0 | 1 | 0 | 0 | 1 | 1 | 75% |
| *ospD* | | 1 | 1 | ab. | 1 | ab. | ab. | ab. | 1 | 0 | 1 | 0 | 1 | 75% |
| P37 | | 0 | 0 | ab. | ab. | ab. | ab. | ab. | 1 | ab. | ab. | 1 | 1 | 60% |
| C6 | | 1 | 1 | 1 | 1 | 1 | ab. | 1 | ab. | 1 | 1 | 1 | ab. | 100% |
| P45-13 | | 1 | 1 | 1 | 0 | 1 | 1 | 0 | 1 | 1 | 1 | 0 | ab. | 73% |
| Congruence score | | 55% | 73% | 78% | 60% | 56% | 75% | 56% | 100% | 30% | 67% | 55% | 78% |  |

Table S7: Congruence between core genome and plasmid gene phylogenies using the IGS classification method. Congruence was assessed across 12 phylogenetic groups based on monophyly and haplotype correspondence. “ab.” indicates absence of the gene sequence for the corresponding taxon.

| Plasmid genes | Monophyletic groups (Core genome) | Group1 | Group2 | Group3 | Group4 | Group5 | Group6 | Group7 | Group8 | Group9 | Group10 | Group11 | Group12 | Congruence score |
| --- | --- | --- | --- | --- | --- | --- | --- | --- | --- | --- | --- | --- | --- | --- |
| *dpbA* | | 1 | 1 | 1 | 0 | 0 | 0 | 0 | 1 | 0 | 1 | 0 | 0 | 42% |
| *dpbB* | | 1 | 1 | 0 | 1 | 0 | 0 | 0 | 1 | 0 | 1 | 0 | 1 | 50% |
| Fibronectin P35 | | 0 | 0 | 0 | 0 | 1 | 0 | 0 | 0 | 1 | ab. | 0 | 0 | 18% |
| *oms28* | | 0 | 0 | 1 | 0 | 0 | 0 | 0 | 1 | 0 | 0 | 0 | 1 | 25% |
| *ospA* | | 0 | 0 | 1 | 0 | 1 | 0 | 0 | ab. | 0 | 0 | 0 | 0 | 18% |
| *ospB* | | 0 | 0 | 0 | 0 | 1 | 0 | 0 | 1 | 0 | 1 | 0 | 1 | 33% |
| *ospC* | | 1 | 1 | 1 | 0 | 1 | 0 | 0 | 0 | 0 | 0 | 0 | 1 | 42% |
| *ospD* | | 1 | 1 | ab. | 0 | ab. | ab. | ab. | 1 | 0 | 1 | 0 | 1 | 63% |
| P37 | | 0 | 0 | ab. | ab. | ab. | ab. | ab. | 0 | ab. | ab. | 1 | 1 | 40% |
| C6 | | 1 | 1 | 1 | 0 | 1 | ab. | 0 | ab. | 1 | 1 | 0 | ab. | 67% |
| P45-13 | | 0 | 0 | 1 | 0 | 1 | 0 | 0 | 1 | 0 | 1 | 0 | ab. | 36% |
| Congruence score | | 45% | 45% | 67% | 10% | 67% | 0% | 0% | 67% | 20% | 67% | 9% | 67% |  |

Table S8: Congruence between core genome and plasmid gene phylogenies using the RSP classification method. Congruence was evaluated across 12 phylogenetic groups based on monophyly and haplotype correspondence. “ab.” indicates absence of the gene sequence for the corresponding taxon.

| Plasmid genes | Monophyletic groups (Core genome) | Group1 | Group2 | Group3 | Group4 | Group5 | Group6 | Group7 | Group8 | Group9 | Group10 | Group11 | Group12 | Congruence score |
| --- | --- | --- | --- | --- | --- | --- | --- | --- | --- | --- | --- | --- | --- | --- |
| *dpbA* | | 1 | 1 | 1 | 0 | 0 | 1 | 0 | 1 | 0 | 1 | 1 | 0 | 58% |
| *dpbB* | | 1 | 1 | 1 | 0 | 0 | 1 | 0 | 1 | 0 | 1 | 1 | 1 | 67% |
| Fibronectin P35 | | 1 | 1 | 0 | 1 | 1 | 1 | 0 | 0 | 1 | ab. | 1 | 0 | 64% |
| *oms28* | | 0 | 1 | 1 | 0 | 0 | 0 | 0 | 1 | 0 | 0 | 0 | 1 | 33% |
| *ospA* | | 1 | 1 | 1 | 0 | 1 | 0 | 0 | ab. | 0 | 0 | 0 | 0 | 36% |
| *ospB* | | 1 | 1 | 0 | 1 | 1 | 1 | 0 | 1 | 0 | 1 | 0 | 1 | 67% |
| *ospC* | | 1 | 1 | 1 | 1 | 1 | 1 | 0 | 0 | 0 | 0 | 1 | 1 | 67% |
| *ospD* | | 1 | 1 | ab. | 1 | ab. | ab. | ab. | 1 | 0 | 1 | 0 | 1 | 75% |
| P37 | | 1 | 1 | ab. | ab. | ab. | ab. | ab. | 1 | ab. | ab. | 1 | 1 | 100% |
| C6 | | 1 | 1 | 1 | 1 | 1 | ab. | 0 | ab. | 1 | 1 | 0 | ab. | 78% |
| P45-13 | | 1 | 1 | 1 | 0 | 1 | 1 | 0 | 1 | 0 | 1 | 0 | ab. | 64% |
| Congruence score | | 91% | 100% | 78% | 50% | 67% | 75% | 0% | 78% | 20% | 67% | 45% | 67% |  |

Table S9: Congruence between core genome and plasmid gene phylogenies using the RST classification method. Congruence was evaluated across 12 phylogenetic groups based on monophyly and haplotype correspondence. “ab.” indicates absence of the gene sequence for the corresponding taxon.

| Plasmid genes | Monophyletic groups (Core genome) | Group1 | Group2 | Group3 | Group4 | Group5 | Group6 | Group7 | Group8 | Group9 | Group10 | Group11 | Group12 | Congruence score |
| --- | --- | --- | --- | --- | --- | --- | --- | --- | --- | --- | --- | --- | --- | --- |
| *dpbA* | | 1 | 1 | 0 | 0 | 0 | 0 | 0 | 0 | 0 | 0 | 0 | 0 | 17% |
| *dpbB* | | 1 | 1 | 0 | 0 | 0 | 0 | 0 | 0 | 0 | 0 | 1 | 1 | 33% |
| Fibronectin P35 | | 0 | 0 | 0 | 0 | 0 | 0 | 0 | 0 | 0 | ab. | 0 | 0 | 0% |
| *oms28* | | 0 | 1 | 0 | 0 | 0 | 0 | 0 | 0 | 0 | 0 | 0 | 0 | 8% |
| *ospA* | | 0 | 0 | 0 | 0 | 0 | 0 | 0 | ab. | 0 | 0 | 0 | 0 | 0% |
| *ospB* | | 0 | 0 | 0 | 0 | 0 | 0 | 0 | 0 | 0 | 0 | 0 | 0 | 0% |
| *ospC* | | 1 | 1 | 0 | 0 | 0 | 0 | 0 | 0 | 0 | 0 | 0 | 0 | 17% |
| *ospD* | | 1 | 1 | ab. | 0 | ab. | ab. | ab. | 0 | 0 | 0 | 0 | 0 | 25% |
| P37 | | 0 | 0 | ab. | ab. | ab. | ab. | ab. | 0 | ab. | ab. | 0 | 0 | 0% |
| C6 | | 1 | 1 | 0 | 0 | 0 | ab. | 0 | ab. | 0 | 0 | 0 | ab. | 22% |
| P45-13 | | 0 | 0 | 0 | 0 | 0 | 0 | 0 | 0 | 0 | 0 | 0 | ab. | 0% |
| Congruence score | | 45% | 55% | 0% | 0% | 0% | 0% | 0% | 0% | 0% | 0% | 9% | 11% |  |

Table S10: Summary of seven monophyletic groups of *Borrelia burgdorferi* sensu stricto identified in this study (*) and their corresponding reference strains with whole-genome data from GenBank. Groups were characterized using five genotyping methods (ospC major group [MG], MLST, IGS, RSP, and RST). “WGS” indicates the availability of whole-genome sequences; “ab.” denotes absence of data.

| Monophyletic group* | Strains | *ospC* MG | MLST | IGS | RSP | RST | Host | Geographic location | WGS (yes/no) | Year | References |
| --- | --- | --- | --- | --- | --- | --- | --- | --- | --- | --- | --- |
| Group 1 (ST55, IGS-1A, RSP2, RST1) [*ospA*] | UWI242 | A | 55 | 1A | 2 | 1 | Human | Wisconsin, USA | yes | 1998 | Lemieux et al., 2023 |
|  | UWI261 | A | 55 | 1A | 2 | 1 | Human | Wisconsin, USA | yes | 1999 | Lemieux et al., 2023 |
|  | UWI272 | A | 55 | 1A | 2 | 1 | Human | Wisconsin, USA | yes | 2001 | Lemieux et al., 2023 |
|  | S90 | A | 55 | 1A | 2 | 1 | I. scapularis on dog | British Columbia Island, Canada | yes |  | Russel et al., 2024 |
| Group 2 (ST1, IGS-1A, RSP1, RST1) [*ospA*] | B31 | A | 1 | 1A | 1 | 1 | Tick | New York, USA | yes | 1981 | Fraser et al., 1997 |
|  | B31_NRZ | A | 1 | 1A | 1 | 1 | Tick | New York, USA | yes | 1981 | Margos et al., 2017 |
|  | PAbe | A | 1 | 1A | 1 | 1 | Human | Munich, Germany | yes | 1997 | Margos et al., 2017 |
|  | PAli | A | 1 | 1A | 1 | 1 | Human | Regensburg, Germany | yes | 1994 | Margos et al., 2017 |
|  | FDAARGOS_196 | A | 1 | 1A | 1 | 1 | Tick | Maryland, USA | yes | 1981 | Kerrigan et al., 2018 (unpublished) |
|  | B31-5A4 | A | 1 | 1A | 1 | 1 | Tick | New York, USA | yes | 1981 | Combs et al., 2022 |
|  | P1286 | A | 1 | 1A | 1 | 1 | NA | NA | yes | NA | Piot Anthony, 2024 |
|  | Am293 | A | 1 | 1A | 1 | 1 | Human | Washington, USA? | yes | 2016 | Mongodin, 2018 |
|  | Am388 | A | 1 | 1A | 1 | 1 | Human | Washington, USA? | yes | 2016 | Mongodin, 2018 |
|  | UMA8 | A | 1 | 1A | 1 | 1 | Human | Connecticut, USA | yes | 2015 | Lemieux et al., 2023 |
|  | UMA9 | A | 1 | 1A | 1 | 1 | Human | Connecticut, USA | yes | 2015 | Lemieux et al., 2023 |
|  | UMA12 | A | 1 | 1A | 1 | 1 | Human | Connecticut, USA | yes | 2015 | Lemieux et al., 2023 |
|  | UMA14 | A | 1 | 1A | 1 | 1 | Human | Connecticut, USA | yes | 2015 | Lemieux et al., 2023 |
|  | URI24 | A | 1 | 1A | 1 | 1 | Human | Connecticut, USA | yes | 2015 | Lemieux et al., 2023 |
|  | UCT29 | A | 1 | 1A | 1 | 1 | Human | Connecticut, USA | yes | 1999 | Lemieux et al., 2023 |
|  | UCT31 | A | 1 | 1A | 1 | 1 | Human | Connecticut, USA | yes | 1999 | Lemieux et al., 2023 |
|  | URI40 | A | 1 | 1A | 1 | 1 | Human | Connecticut, USA | yes | 1999 | Lemieux et al., 2023 |
|  | URI41 | A | 1 | 1A | 1 | 1 | Human | Connecticut, USA | yes | 1999 | Lemieux et al., 2023 |
|  | URI42 | A | 1 | 1A | 1 | 1 | Human | Connecticut, USA | yes | 1999 | Lemieux et al., 2023 |
|  | URI43 | A | 1 | 1A | 1 | 1 | Human | Connecticut, USA | yes | 1999 | Lemieux et al., 2023 |
|  | URI61 | A | 1 | 1A | 1 | 1 | Human | Connecticut, USA | yes | 1999 | Lemieux et al., 2023 |
|  | URI63 | A | 1 | 1A | 1 | 1 | Human | Connecticut, USA | yes | 1999 | Lemieux et al., 2023 |
|  | URI65 | A | 1 | 1A | 1 | 1 | Human | Connecticut, USA | yes | 1999 | Lemieux et al., 2023 |
|  | UCT66 | A | 1 | 1A | 1 | 1 | Human | Connecticut, USA | yes | 1999 | Lemieux et al., 2023 |
|  | URI72 | A | 1 | 1A | 1 | 1 | Human | Connecticut, USA | yes | 2000 | Lemieux et al., 2023 |
|  | UCT74 | A | 1 | 1A | 1 | 1 | Human | Connecticut, USA | yes | 2000 | Lemieux et al., 2023 |
|  | URI75 | A | 1 | 1A | 1 | 1 | Human | Connecticut, USA | yes | 2000 | Lemieux et al., 2023 |
|  | URI89 | A | 1 | 1A | 1 | 1 | Human | Connecticut, USA | yes | 2000 | Lemieux et al., 2023 |
|  | URI91 | A | 1 | 1A | 1 | 1 | Human | Connecticut, USA | yes | 2000 | Lemieux et al., 2023 |
|  | URI93 | A | 1 | 1A | 1 | 1 | Human | Connecticut, USA | yes | 2000 | Lemieux et al., 2023 |
|  | URI101 | A | 1 | 1A | 1 | 1 | Human | Connecticut, USA | yes | 1998 | Lemieux et al., 2023 |
|  | URI102 | A | 1 | 1A | 1 | 1 | Human | Connecticut, USA | yes | 1998 | Lemieux et al., 2023 |
|  | URI105 | A | 1 | 1A | 1 | 1 | Human | Connecticut, USA | yes | 2000 | Lemieux et al., 2023 |
|  | URI107 | A | 1 | 1A | 1 | 1 | Human | Connecticut, USA | yes | 2000 | Lemieux et al., 2023 |
|  | UCT109 | A | 1 | 1A | 1 | 1 | Human | Connecticut, USA | yes | 2000 | Lemieux et al., 2023 |
|  | URI111 | A | 1 | 1A | 1 | 1 | Human | Connecticut, USA | yes | 2001 | Lemieux et al., 2023 |
|  | URI119 | A | 1 | 1A | 1 | 1 | Human | Connecticut, USA | yes | 2001 | Lemieux et al., 2023 |
|  | URI120 | A | 1 | 1A | 1 | 1 | Human | Connecticut, USA | yes | 2001 | Lemieux et al., 2023 |
|  | UNY129 | A | 1 | 1A | 1 | 1 | Human | New York, USA | yes | 1992 | Lemieux et al., 2023 |
|  | UNY145 | A | 1 | 1A | 1 | 1 | Human | New York, USA | yes | 1995 | Lemieux et al., 2023 |
|  | UNY153 | A | 1 | 1A | 1 | 1 | Human | New York, USA | yes | 1995 | Lemieux et al., 2023 |
|  | UNY161 | A | 1 | 1A | 1 | 1 | Human | New York, USA | yes | 1996 | Lemieux et al., 2023 |
|  | UNY170 | A | 1 | 1A | 1 | 1 | Human | New York, USA | yes | 1997 | Lemieux et al., 2023 |
|  | UNY184 | A | 1 | 1A | 1 | 1 | Human | New York, USA | yes | 1998 | Lemieux et al., 2023 |
|  | UNY192 | A | 1 | 1A | 1 | 1 | Human | New York, USA | yes | 2000 | Lemieux et al., 2023 |
|  | UNY195 | A | 1 | 1A | 1 | 1 | Human | New York, USA | yes | 2000 | Lemieux et al., 2023 |
|  | UNY200 | A | 1 | 1A | 1 | 1 | Human | New York, USA | yes | 1991 | Lemieux et al., 2023 |
|  | URI219 | A | 1 | 1A | 1 | 1 | Human | Connecticut, USA | yes | 2015 | Lemieux et al., 2023 |
| Group 3 (ST46, IGS-8C, RSP13, RST3) [*ospC* T1/T] | 1476702 | T | 29 | 8C | 13 | 3 | Tick | Wisconsin, USA | non | 2004 | Travinsky et al.,2010 |
| Group 5 (ST3, IGS-2A, RSP3, RST2) [*ospC* k] | UMA4 | K | 3 | 2A | 3 | 2 | Human | Connecticut, USA | yes | 2015 | Lemieux et al., 2023 |
|  | URI23 | K | 3 | 2A | 3 | 2 | Human | Connecticut, USA | yes | 2015 | Lemieux et al., 2023 |
|  | UCT30 | K | 3 | 2A | 3 | 2 | Human | Connecticut, USA | yes | 1999 | Lemieux et al., 2023 |
|  | UCT32 | K | 3 | 2A | 3 | 2 | Human | Connecticut, USA | yes | 1999 | Lemieux et al., 2023 |
|  | URI44 | K | 3 | 2A | 3 | 2 | Human | Connecticut, USA | yes | 1999 | Lemieux et al., 2023 |
|  | URI46 | K | 3 | 2A | 3 | 2 | Human | Connecticut, USA | yes | 1999 | Lemieux et al., 2023 |
|  | URI47 | K | 3 | 2A | 3 | 2 | Human | Connecticut, USA | yes | 1999 | Lemieux et al., 2023 |
|  | UCT62 | K | 3 | 2A | 3 | 2 | Human | Connecticut, USA | yes | 1999 | Lemieux et al., 2023 |
|  | URI64 | K | 3 | 2A | 3 | 2 | Human | Connecticut, USA | yes | 2000 | Lemieux et al., 2023 |
|  | UCT73 | K | 3 | 2A | 3 | 2 | Human | Connecticut, USA | yes | 2000 | Lemieux et al., 2023 |
|  | UCT76 | K | 3 | 2A | 3 | 2 | Human | Connecticut, USA | yes | 2000 | Lemieux et al., 2023 |
|  | URI77 | K | 3 | 2A | 3 | 2 | Human | Connecticut, USA | yes | 2000 | Lemieux et al., 2023 |
|  | UCT83 | K | 3 | 2A | 3 | 2 | Human | Connecticut, USA | yes | 2000 | Lemieux et al., 2023 |
|  | UCT92 | K | 3 | 2A | 3 | 2 | Human | Connecticut, USA | yes | 2000 | Lemieux et al., 2023 |
|  | URI94 | K | 3 | 2A | 3 | 2 | Human | Connecticut, USA | yes | 2001 | Lemieux et al., 2023 |
|  | URI97 | K | 3 | 2A | 3 | 2 | Human | Connecticut, USA | yes | 1998 | Lemieux et al., 2023 |
|  | URI98 | K | 3 | 2A | 3 | 2 | Human | Connecticut, USA | yes | 1998 | Lemieux et al., 2023 |
|  | URI103 | K | 3 | 2A | 3 | 2 | Human | Connecticut, USA | yes | 1998 | Lemieux et al., 2023 |
|  | UCT104 | K | 3 | 2A | 3 | 2 | Human | Connecticut, USA | yes | 2000 | Lemieux et al., 2023 |
|  | UCT110 | K | 3 | 2A | 3 | 2 | Human | Connecticut, USA | yes | 2000 | Lemieux et al., 2023 |
|  | URI112 | K | 3 | 2A | 3 | 2 | Human | Connecticut, USA | yes | 2001 | Lemieux et al., 2023 |
|  | URI117 | K | 3 | 2A | 3 | 2 | Human | Connecticut, USA | yes | 2001 | Lemieux et al., 2023 |
|  | URI118 | K | 3 | 2A | 3 | 2 | Human | Connecticut, USA | yes | 2001 | Lemieux et al., 2023 |
|  | URI122 | K | 3 | 2A | 3 | 2 | Human | Connecticut, USA | yes | 2001 | Lemieux et al., 2023 |
|  | UNY130 | K | 3 | 2A | 3 | 2 | Human | New York, USA | yes | 1992 | Lemieux et al., 2023 |
|  | UNY146 | K | 3 | 2A | 3 | 2 | Human | New York, USA | yes | 1995 | Lemieux et al., 2023 |
|  | UNY149 | K | 3 | 2A | 3 | 2 | Human | New York, USA | yes | 1995 | Lemieux et al., 2023 |
|  | UNY154 | K | 3 | 2A | 3 | 2 | Human | New York, USA | yes | 1995 | Lemieux et al., 2023 |
|  | UNY155 | K | 3 | 2A | 3 | 2 | Human | New York, USA | yes | 1995 | Lemieux et al., 2023 |
|  | UNY156 | K | 3 | 2A | 3 | 2 | Human | New York, USA | yes | 1995 | Lemieux et al., 2023 |
|  | UNY160 | K | 3 | 2A | 3 | 2 | Human | New York, USA | yes | 1999 | Lemieux et al., 2023 |
|  | UNY177 | K | 3 | 2A | 3 | 2 | Human | New York, USA | yes | 1997 | Lemieux et al., 2023 |
|  | UNY179 | K | 3 | 2A | 3 | 2 | Human | New York, USA | yes | 1998 | Lemieux et al., 2023 |
|  | UNY180 | K | 3 | 2A | 3 | 2 | Human | New York, USA | yes | 1998 | Lemieux et al., 2023 |
|  | UNY181 | K | 3 | 2A | 3 | 2 | Human | New York, USA | yes | 1998 | Lemieux et al., 2023 |
|  | UNY182 | K | 3 | 2A | 3 | 2 | Human | New York, USA | yes | 1998 | Lemieux et al., 2023 |
|  | UNY185 | K | 3 | 2A | 3 | 2 | Human | New York, USA | yes | 1998 | Lemieux et al., 2023 |
|  | UNY188 | K | 3 | 2A | 3 | 2 | Human | New York, USA | yes | 1999 | Lemieux et al., 2023 |
|  | UNY194 | K | 3 | 2A | 3 | 2 | Human | New York, USA | yes | 2000 | Lemieux et al., 2023 |
|  | UNY197 | K | 3 | 2A | 3 | 2 | Human | New York, USA | yes | 2000 | Lemieux et al., 2023 |
|  | UNY211 | K | 3 | 2A | 3 | 2 | Human | New York, USA | yes | 2002 | Lemieux et al., 2023 |
|  | UNY212 | K | 3 | 2A | 3 | 2 | Human | New York, USA | yes | 2003 | Lemieux et al., 2023 |
|  | B379 | K | 3 | 2A | 3 | 2 | Human | New York, USA | yes | 1994 | Combs et al., 2022 |
|  | 297 | K | 3 | 2A | 3 | 2 | Human | Connecticut, USA | yes |  | Piot Anthony, 2024 |
|  | Sh-2-82 | K | 3 | 2A | 3 | 2 | Human | Washington, USA? | yes | 2016 | Mongodin, 2018 |
| Group 7 (ST29, IGS-2D, RSP5, RST2) [*ospC* A3/L] | UWI234 | L | 29 | 2D | 5 | 2 | Human | US Midwest | yes | 1997 | Lemieux et al., 2023 |
|  | UWI238 | L | 29 | 2D | 5 | 2 | Human | US Midwest | yes | 1997 | Lemieux et al., 2023 |
|  | UWI252 | L | 29 | 2D | 5 | 2 | Human | US Midwest | yes | 1998 | Lemieux et al., 2023 |
|  | UWI256 | L | 29 | 2D | 5 | 2 | Human | US Midwest | yes | 1999 | Lemieux et al., 2023 |
|  | UWI263 | L | 29 | 2D | 5 | 2 | Human | US Midwest | yes | 1999 | Lemieux et al., 2023 |
|  | UWI264 | L | 29 | 2D | 5 | 2 | Human | US Midwest | yes | 2000 | Lemieux et al., 2023 |
|  | S11 | L | 29 | 2D | 5 | 2 | Tick (I. pacificus) | British Columbia, Canada | yes | 1994 | Russel et al., 2024 |
|  | S48 | L | 29 | 2D | 5 | 2 | I. pacificus on Dog | British Columbia, Canada | yes | 2010 | Russel et al., 2024 |
|  | GMT-0122 | L | 29 | 2D | 5 | 2 | NA | NA | yes | NA | Piot Anthony, 2024 |
| Group 8 (ST530, IGS-4A, RSP6, RST3) [*ospC* C3] | BRF-226 | C3 | 530 | 4A | 6 | 3 | Tick | Wisconsin, USA | yes | 2013 | Carpi et al., 2015 |
| Group 11 (ST43, IGS-5, RSP14 , RST3) [*ospC* N] | UWI229 | N | 43 | 5 | 14 | 3 | Human | US Midwest | yes | 1996 | Lemieux et al., 2023 |
|  | S04 | N | 43 | 5 | 14 | 3 | I. angustus on mouse | British Columbia, Canada | yes | 1993 | Russel et al., 2024 |

Fig. S1. Maximum likelihood phylogenetic tree of Borrelia burgdorferi sensu stricto strains inferred using a partitioned GTR model. Independent evolutionary rates were applied for each partition (*BmpA*, *FlaB*, *oms66*, P83-100, eight housekeeping genes, and the 16S–23S marker). Twelve phylogenetic groups are indicated, with branch support values shown as SH-aLRT (Shimodaira–Hasegawa approximate likelihood ratio test) and UFBoot (Ultrafast Bootstrap).

Fig. S2. Maximum likelihood phylogenetic tree of *Borrelia burgdorferi* sensu stricto strains inferred using a concatenated GTR model. A single evolutionary rate was applied across all partitions. Twelve phylogenetic groups are indicated, with branch support values shown as SH-aLRT (Shimodaira–Hasegawa approximate likelihood ratio test) and UFBoot (Ultrafast Bootstrap).


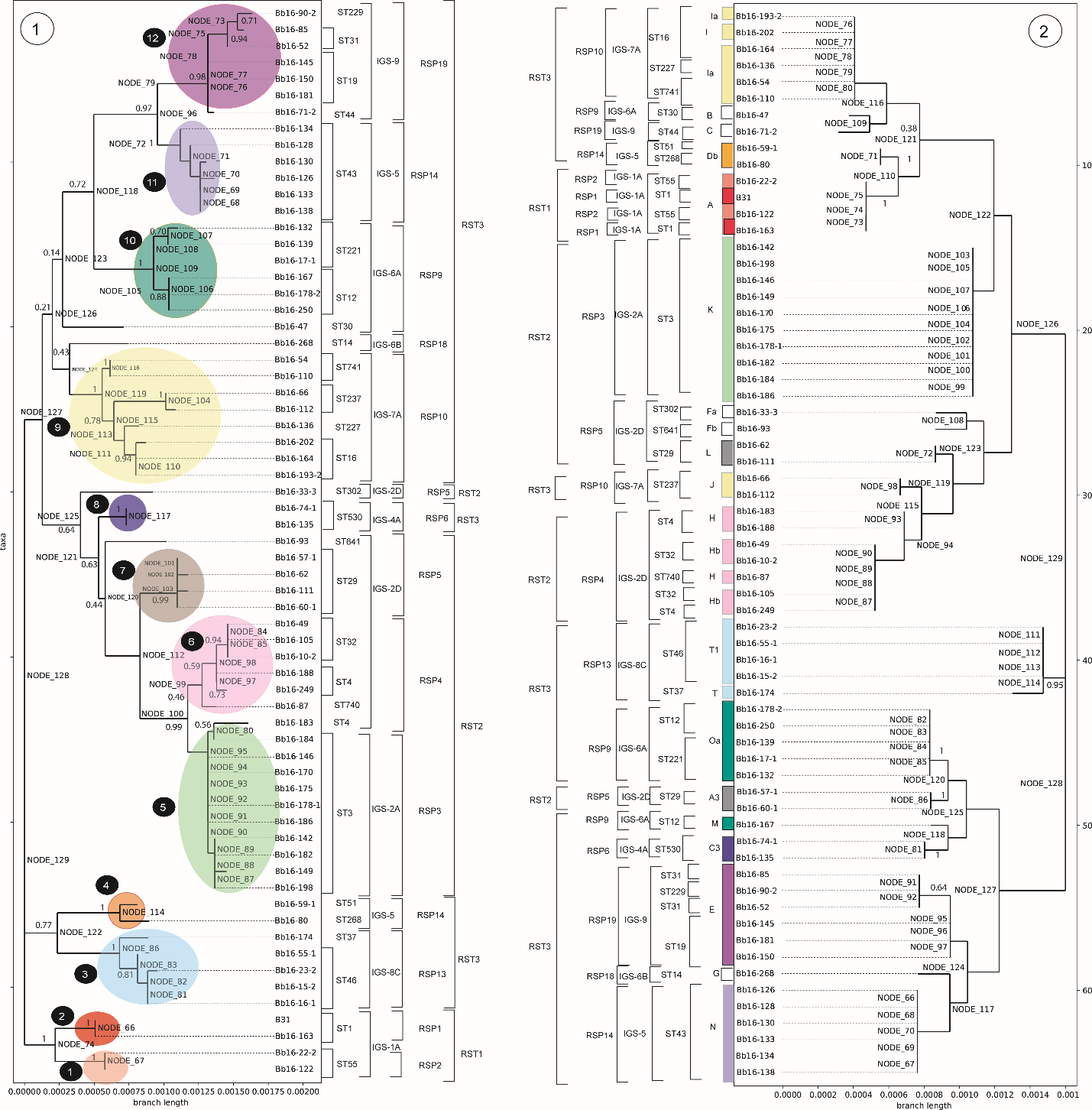


Fig. S3. Comparison of unrooted maximum likelihood (ML) phylogenetic trees of *Borrelia burgdorferi* sensu stricto strains based on the core genome and the *ospC* gene. Twelve monophyletic groups identified in the core genome tree are numbered 1–12, with distinct color coding and circling for clarity. Groups are evaluated for congruence with the *ospC* tree and annotated according to MLST, *ospC* major groups (MG), RSP, and RST typing. Node support values are shown as calculated by ClonalFrameML.


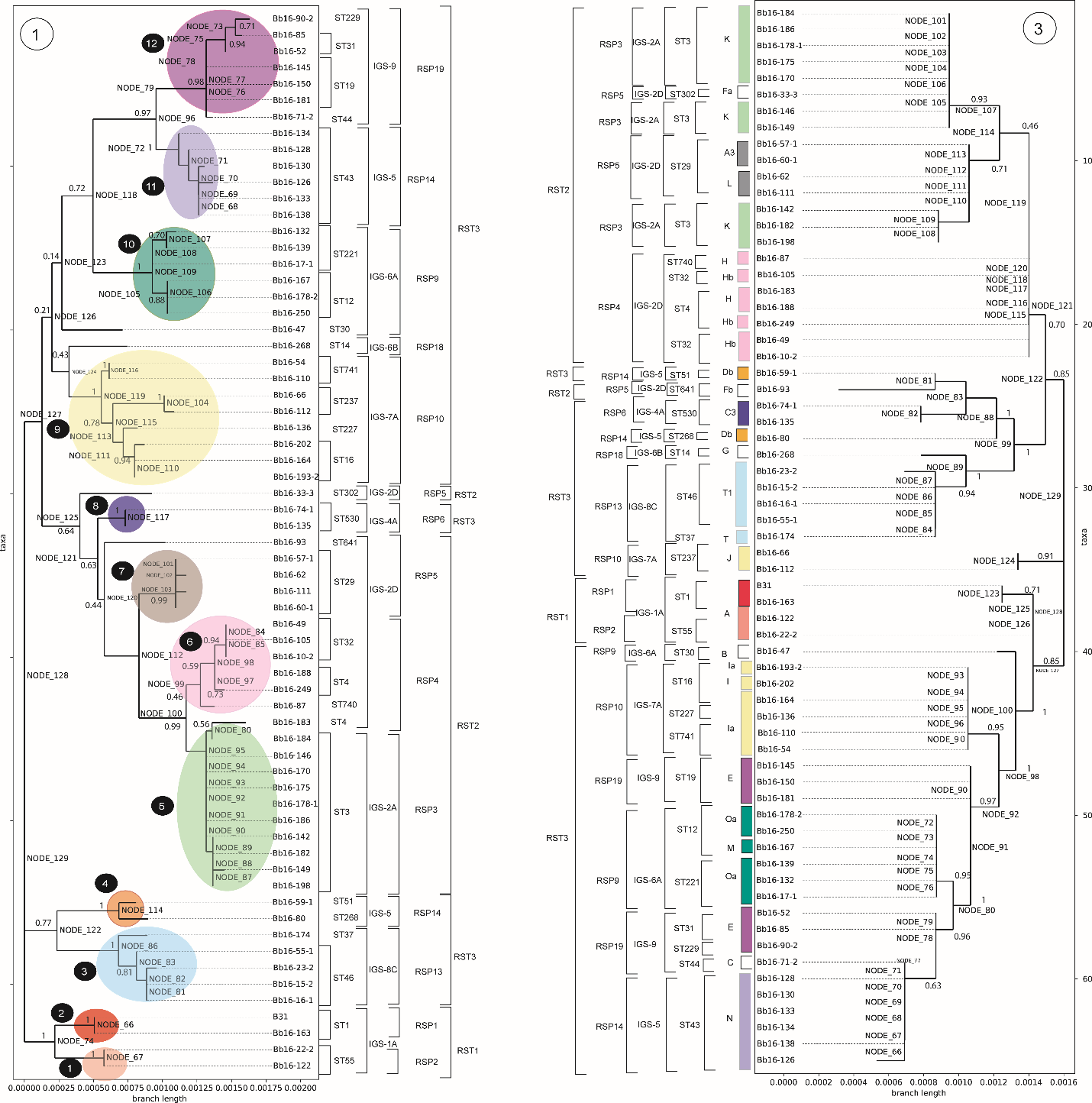


Fig. S4: Comparison of unrooted maximum likelihood (ML) phylogenetic trees of *Borrelia burgdorferi* sensu stricto strains based on the core genome and the *dbpA* gene. Twelve monophyletic groups from the core genome tree are numbered 1–12, with distinct color coding and circling for clarity. Groups are evaluated for congruence with the *dbpA* tree and annotated using MLST, *ospC* major groups (MG), RSP, and RST typing methods. Node support values are shown as calculated by ClonalFrameML.


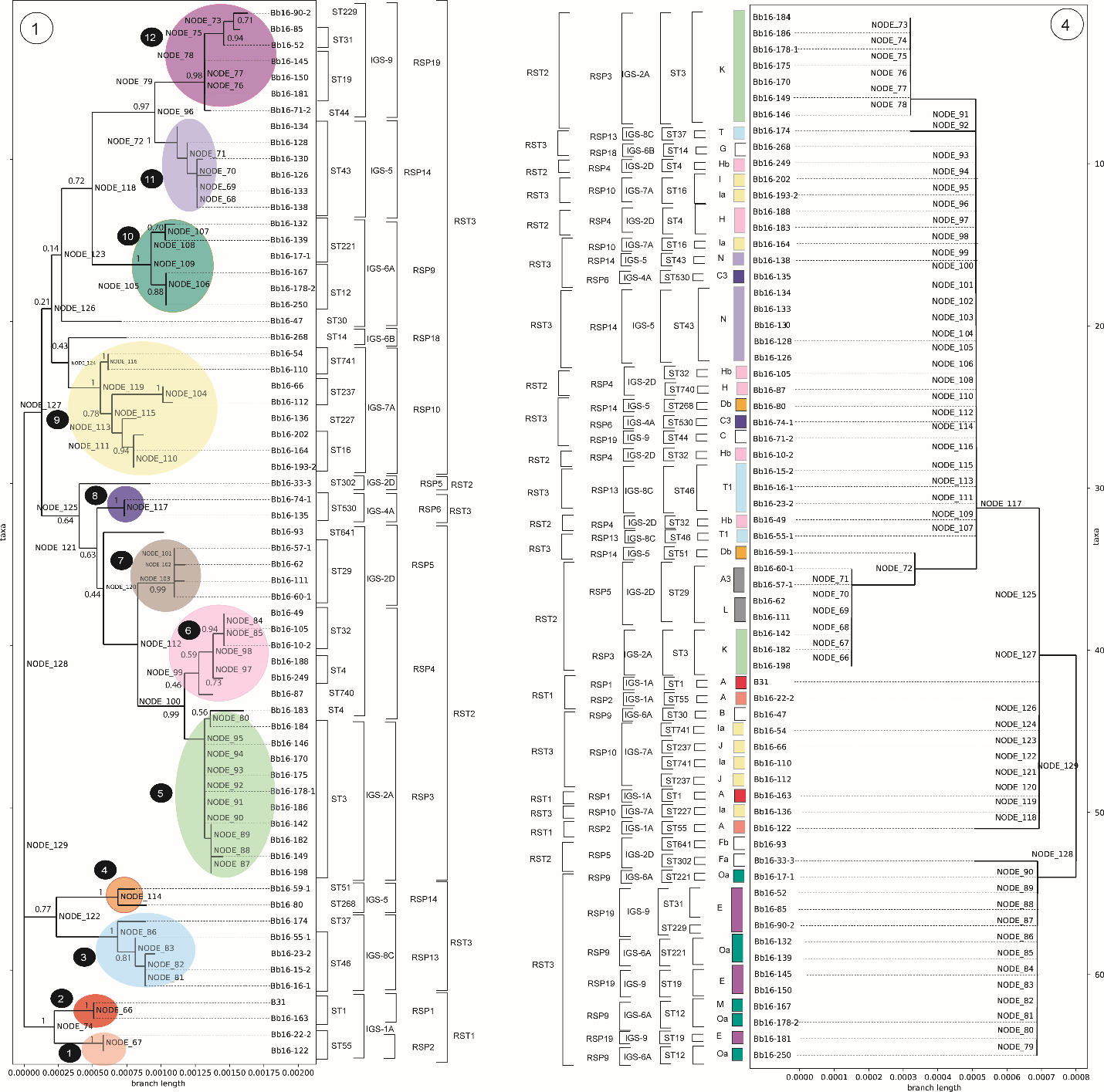


Fig. S5. Comparison of unrooted maximum likelihood (ML) phylogenetic trees of Borrelia burgdorferi sensu stricto strains based on the core genome and the *dbpB* gene. Twelve monophyletic groups from the core genome tree are numbered 1–12, with distinct color coding and circling for clarity. Groups are evaluated for congruence with the *dbpB* tree and annotated using MLST, *ospC* major groups (MG), RSP, and RST typing methods. Node support values are shown as calculated by ClonalFrameML.


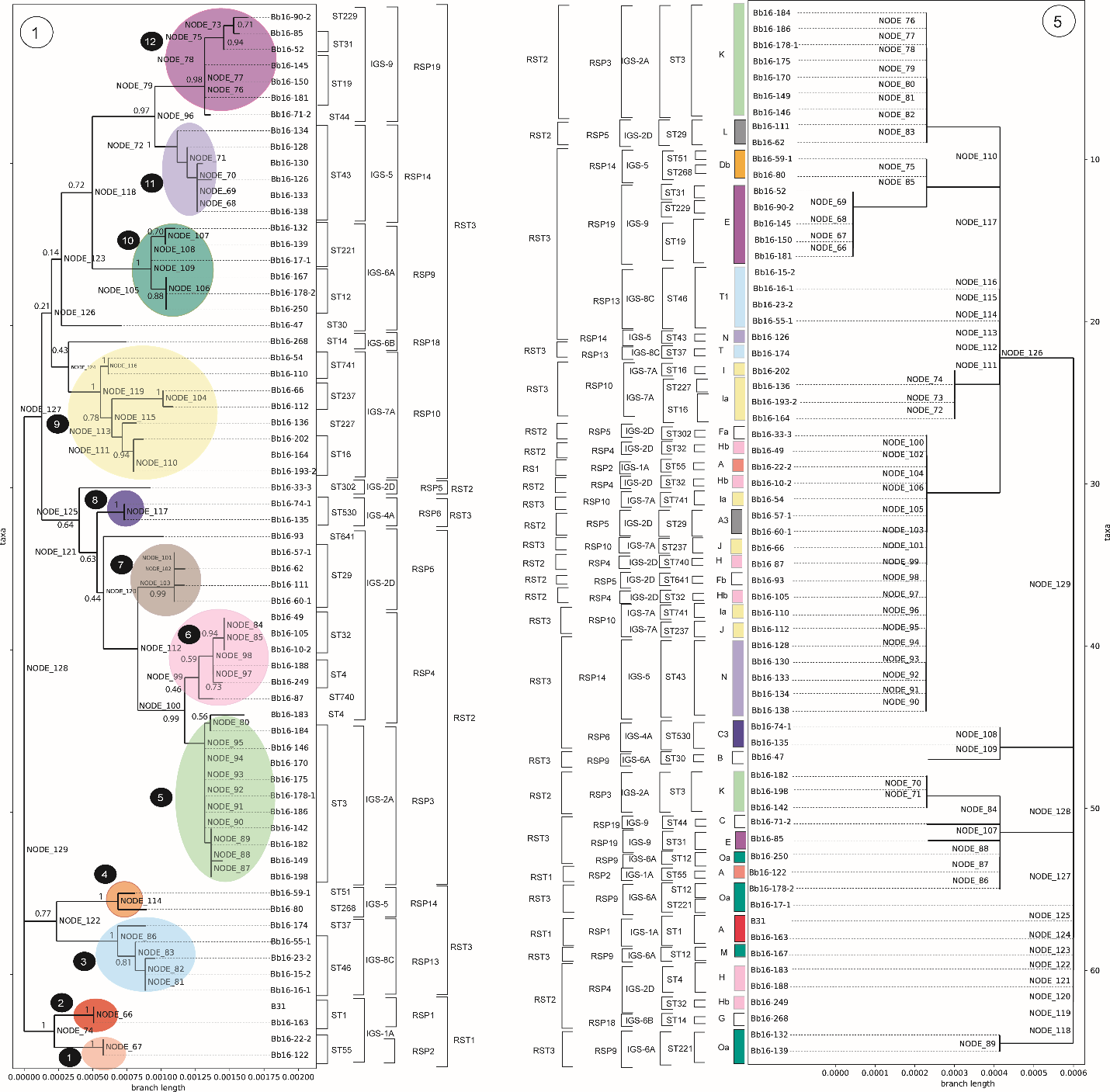


Fig. S6. Comparison of unrooted maximum likelihood (ML) phylogenetic trees of Borrelia burgdorferi sensu stricto strains based on the core genome and the *oms28* gene. Twelve monophyletic groups from the core genome tree are numbered 1–12, with distinct color coding and circling for clarity. Groups are evaluated for congruence with the *oms28* tree and annotated using MLST, *ospC* major groups (MG), RSP, and RST typing methods. Node support values are shown as calculated by ClonalFrameML.


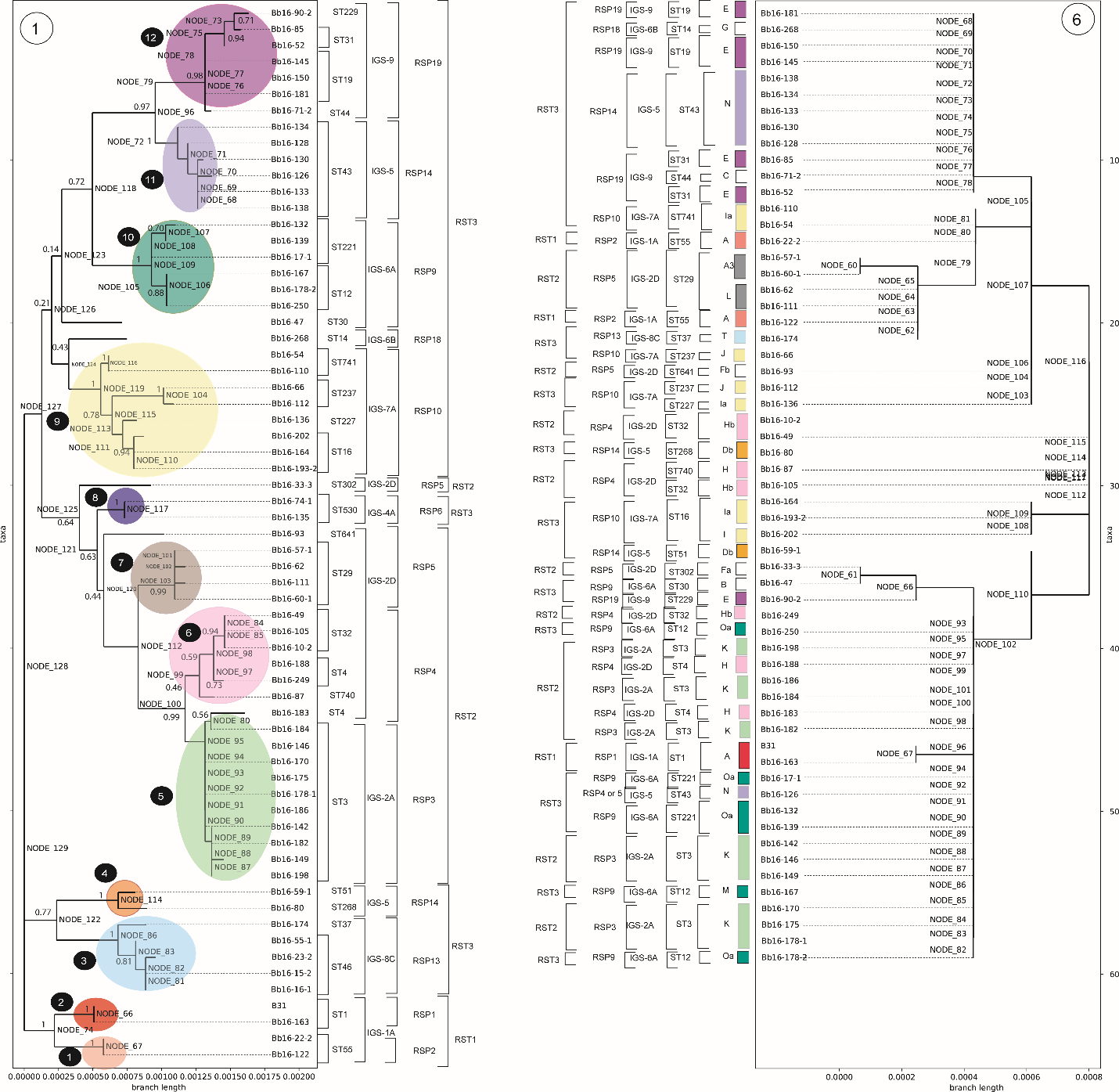


Fig. S7. Comparison of unrooted maximum likelihood (ML) phylogenetic trees of *Borrelia burgdorferi* sensu stricto strains based on the core genome and the *ospA* gene. Twelve monophyletic groups from the core genome tree are numbered 1–12, with distinct color coding and circling for clarity. Groups are evaluated for congruence with the *ospA* tree and annotated using MLST, *ospC* major groups (MG), RSP, and RST typing methods. Node support values are reported as calculated by ClonalFrameML.


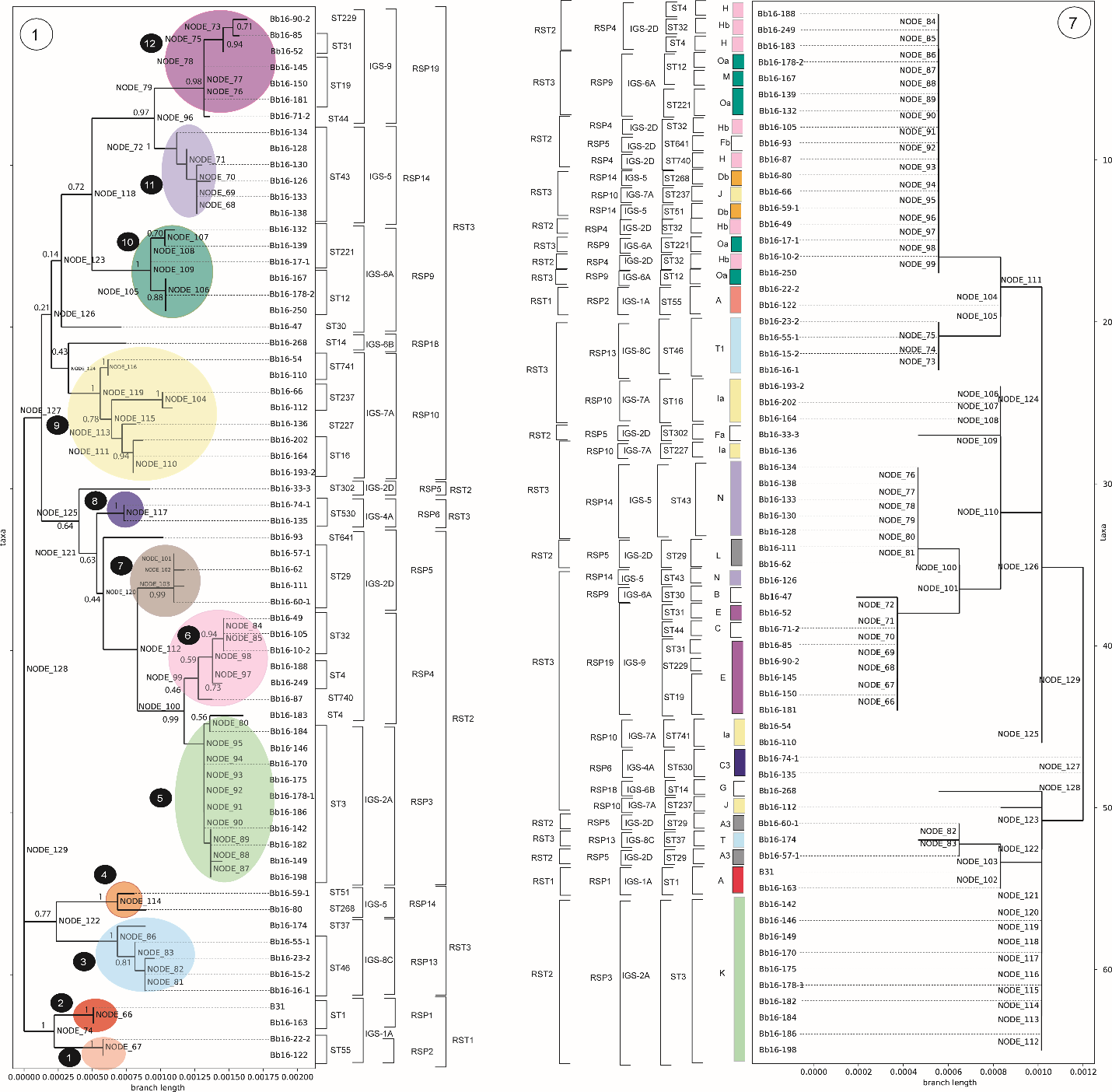


Fig. S8. Comparison of unrooted maximum likelihood (ML) phylogenetic trees of *Borrelia burgdorferi* sensu stricto strains based on the core genome and the *ospB* gene. Twelve monophyletic groups from the core genome tree are numbered 1–12, with distinct color coding and circling for clarity. Groups are evaluated for congruence with the *ospB* tree and annotated using MLST, *ospC* major groups (MG), RSP, and RST typing methods. Node support values are reported as calculated by ClonalFrameML.


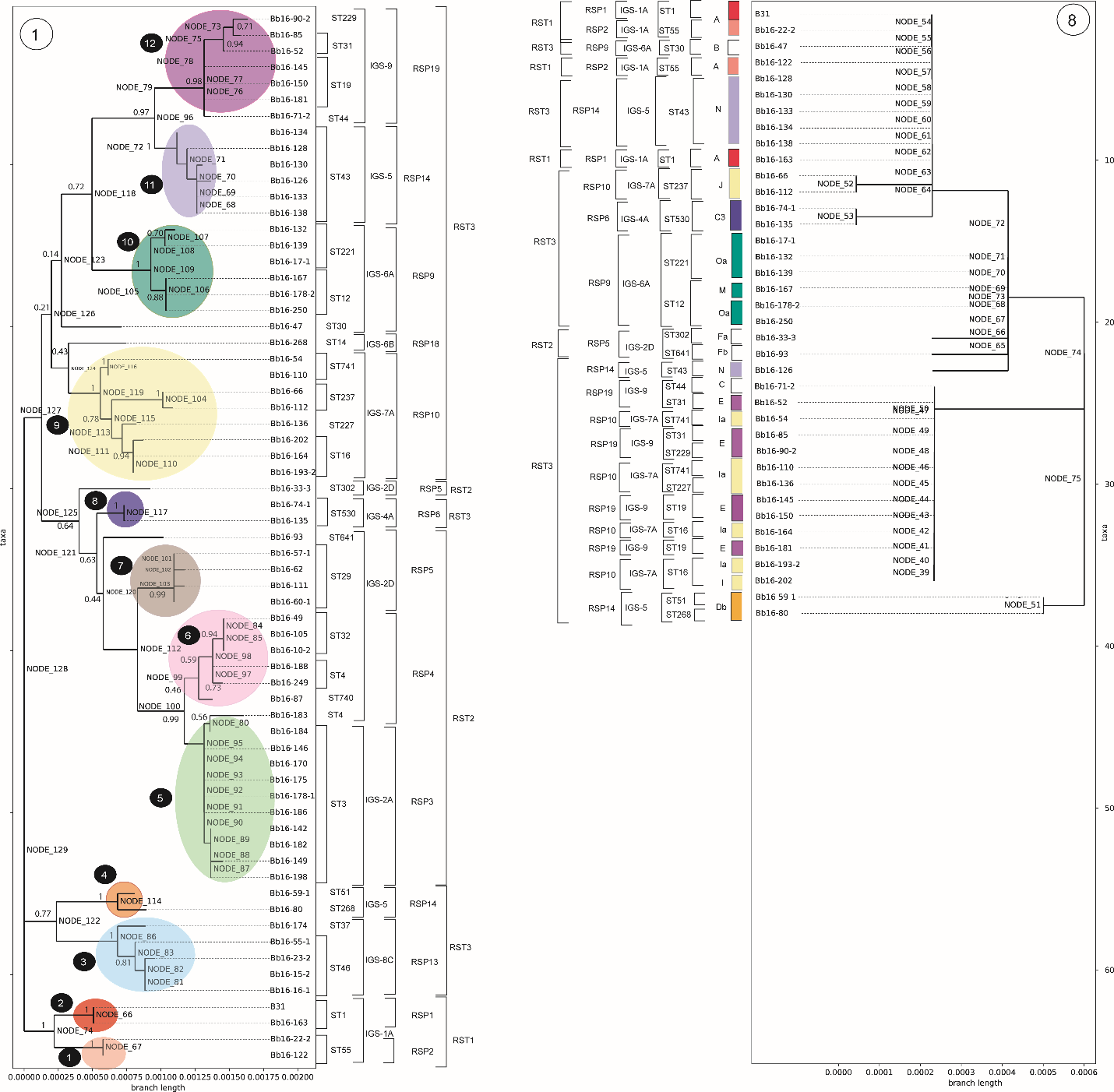


Fig. S9. Comparison of unrooted maximum likelihood (ML) phylogenetic trees of *Borrelia burgdorferi* sensu stricto strains based on the core genome and the *ospD* gene. Twelve monophyletic groups from the core genome tree are numbered 1–12, with distinct color coding and circling for clarity. Groups are evaluated for congruence with the *ospD* tree and annotated using MLST, *ospC* major groups (MG), RSP, and RST typing methods. Node support values are reported as calculated by ClonalFrameML.


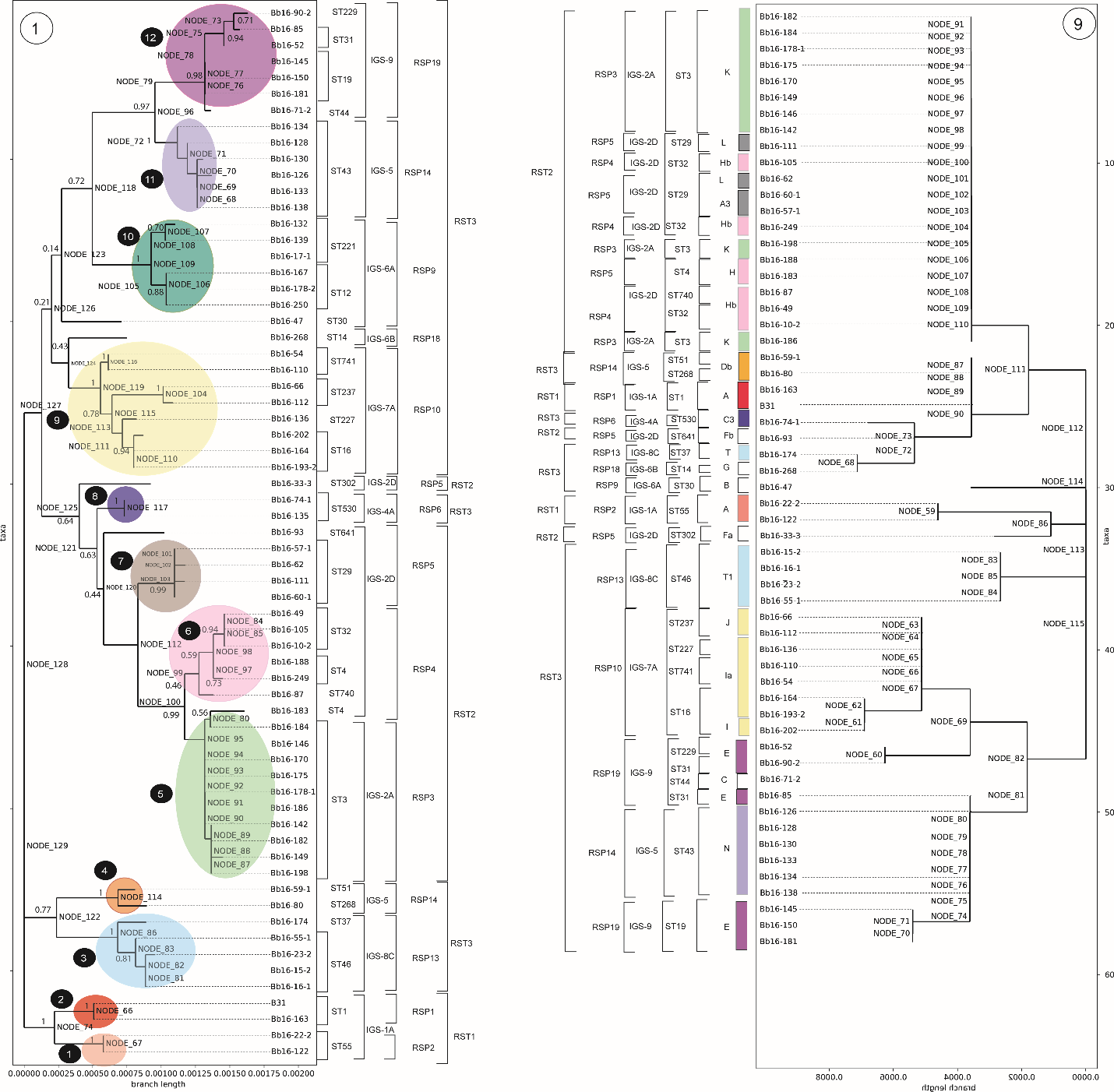


Fig. S10. Comparison of unrooted maximum likelihood (ML) phylogenetic trees of *Borrelia burgdorferi* sensu stricto strains based on the core genome and the fibronectin-binding protein gene (P35). Twelve monophyletic groups from the core genome tree are numbered 1–12, with distinct color coding and circling for clarity. Groups are evaluated for congruence with the fibronectin-binding protein gene tree and annotated using MLST, *ospC* major groups (MG), RSP, and RST typing methods. Node support values are reported as calculated by ClonalFrameML.


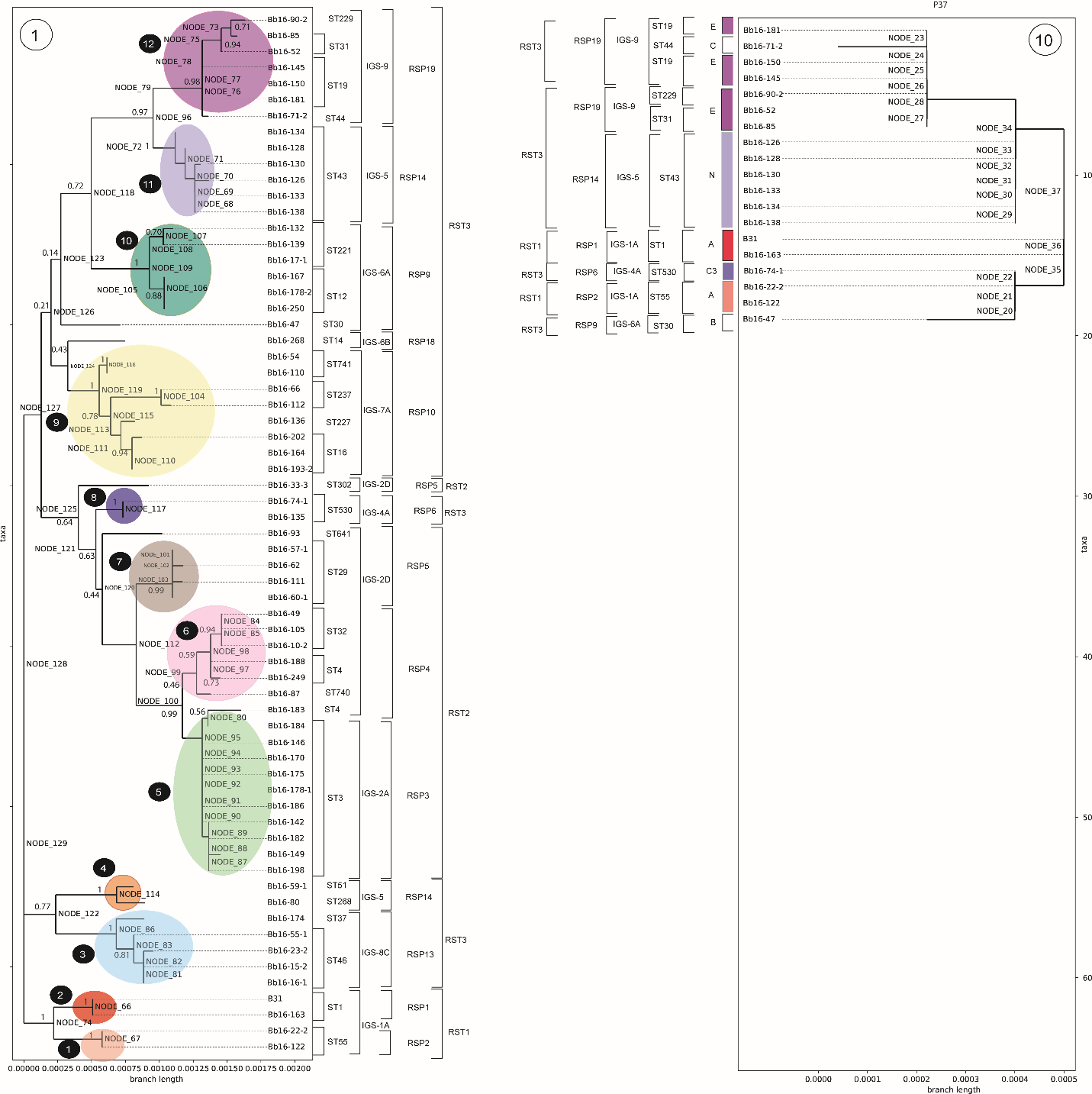


Fig. S11. Comparison of unrooted maximum likelihood (ML) phylogenetic trees of *Borrelia burgdorferi* sensu stricto strains based on the core genome and the P37 gene. Twelve monophyletic groups from the core genome tree are numbered 1–12, with distinct color coding and circling for clarity. Groups are evaluated for congruence with the P37 gene tree and annotated using MLST, *ospC* major groups (MG), RSP, and RST typing methods. Node support values are reported as calculated by ClonalFrameML.


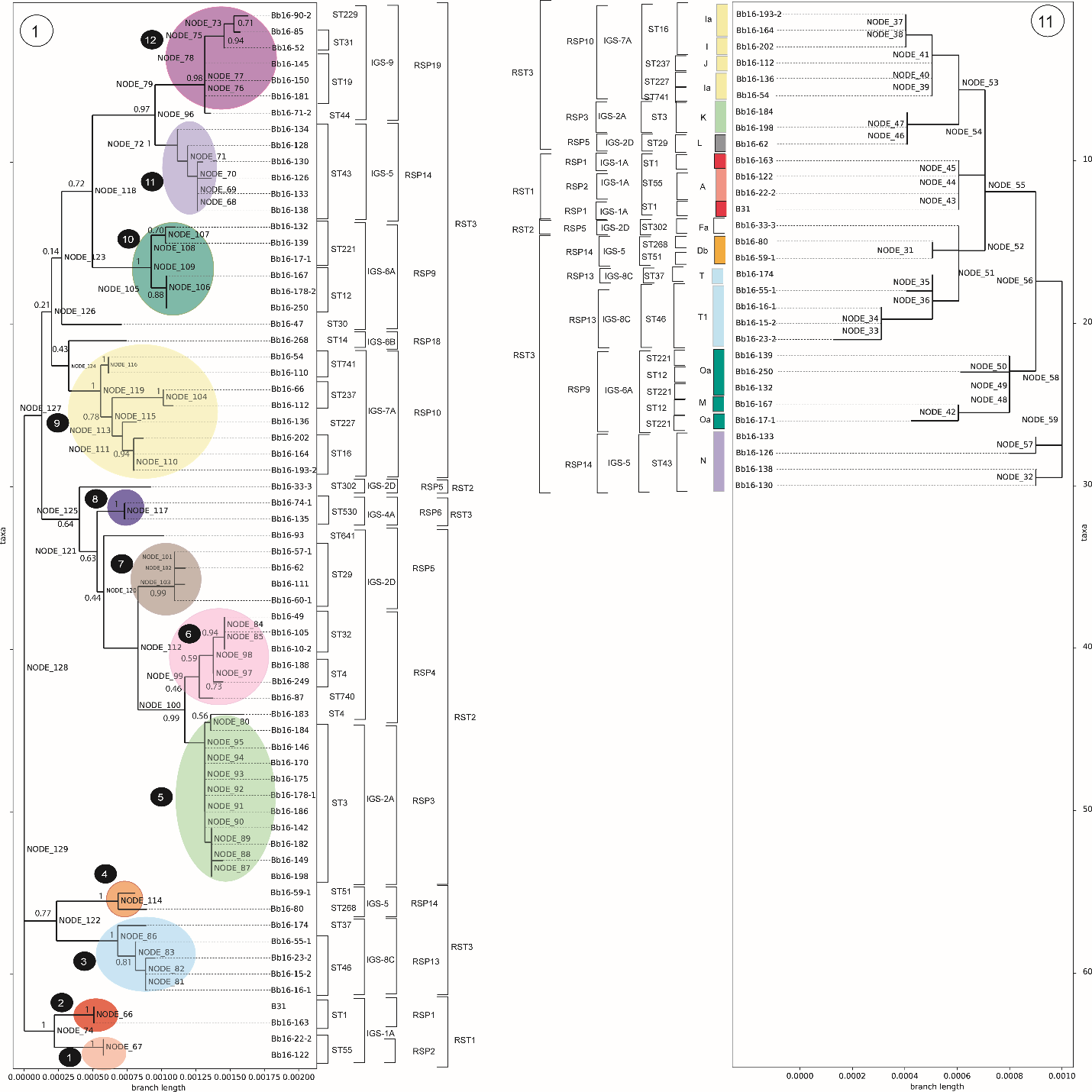


Fig. S12. Comparison of unrooted maximum likelihood (ML) phylogenetic trees of *Borrelia burgdorferi* sensu stricto strains based on the core genome and the C6 peptide of the *VlsE1* gene. Twelve monophyletic groups from the core genome tree are numbered 1–12, with distinct color coding and circling for clarity. Groups are evaluated for congruence with the *VlsE1* (C6 peptide) gene tree and annotated using MLST, *ospC* major groups (MG), RSP, and RST typing methods. Node support values are reported as calculated by ClonalFrameML.


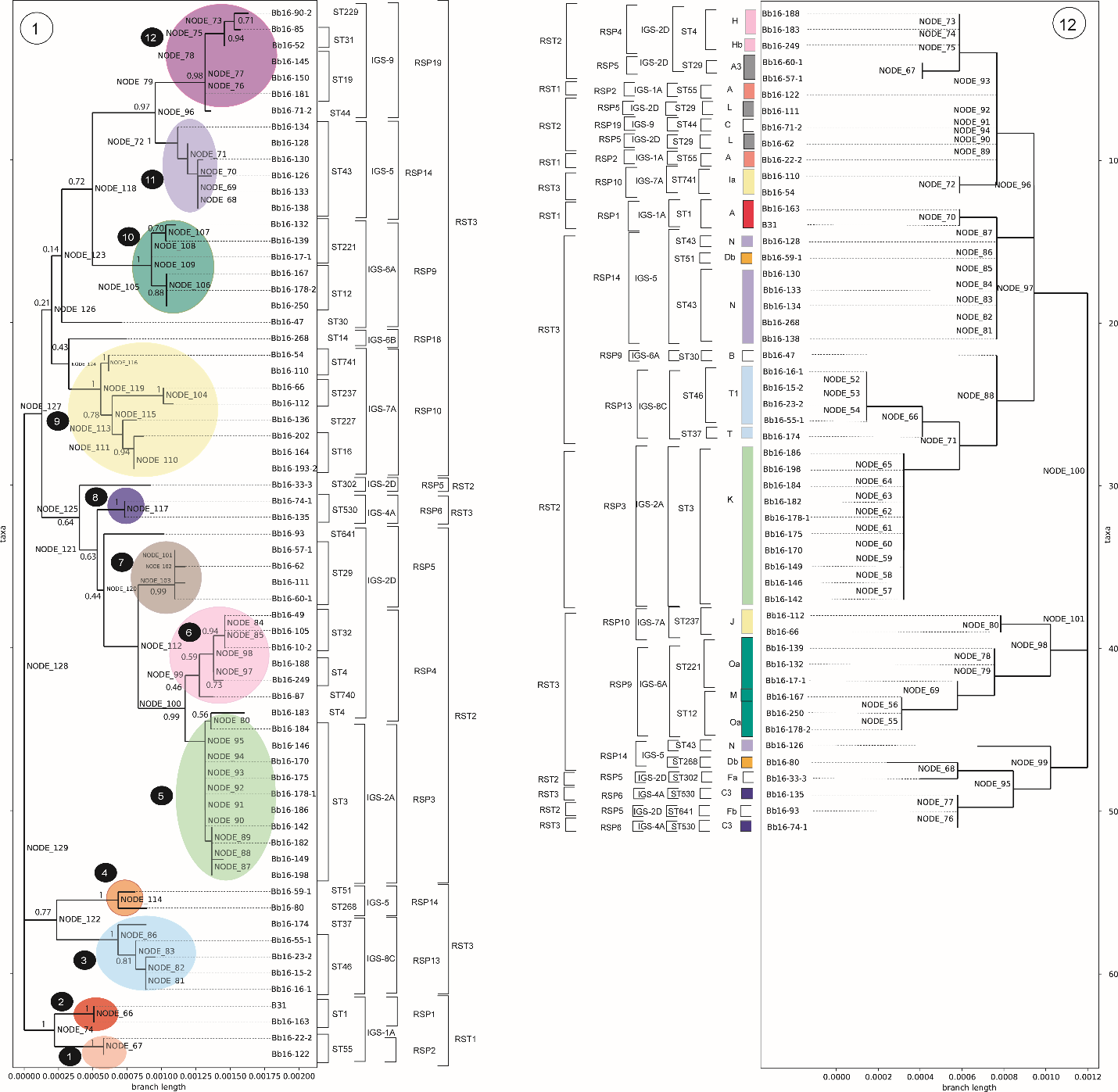


Fig. S13: Comparison of unrooted maximum likelihood (ML) phylogenetic trees between core genome (1) and P45-13 gene (12). The 12 monophyletic groups identified in the core genome phylogeny are numbered from 1 to 12, with distinct color coding and circling for visual clarity. These groups are evaluated for congruence with the *ospC* tree, facilitating comparison. The identification of groups based on MLST, *ospC* major groups (MG), RSP, and RST typing methods is provided. Clade support is further enhanced by reporting node numbers as calculated by ClonalFrameML.

References cited:

1. Zhang, J.-R. & Norris, S. J. Kinetics and In Vivo Induction of Genetic Variation of *vlsE* in *Borrelia burgdorferi*. *Infect. Immun.* **66**, 3689–3697 (1998).

2. Schulte-Spechtel, U., Fingerle, V., Goettner, G., Rogge, S. & Wilske, B. Molecular analysis of decorin-binding protein A (DbpA) reveals five major groups among European Borrelia burgdorferi sensu lato strains with impact for the development of serological assays and indicates lateral gene transfer of the dbpA gene. *Int. J. Med. Microbiol.* **296**, 250–266 (2006).

3. Bontemps-Gallo, S., Lawrence, K. & Gherardini, F. C. Two Different Virulence-Related Regulatory Pathways in Borrelia burgdorferi Are Directly Affected by Osmotic Fluxes in the Blood Meal of Feeding Ixodes Ticks. *PLOS Pathog.* **12**, e1005791 (2016).

4. Bernard, Q. *et al.* Borrelia burgdorferi protein interactions critical for microbial persistence in mammals. *Cell. Microbiol.* **21**, (2019).

5. Fikrig, E. *et al.* Borrelia burgdorferi P35 and P37 Proteins, Expressed In Vivo, Elicit Protective Immunity. *Immunity* **6**, 531–539 (1997).

6. Skare, J. T. *et al.* Porin activity of the native and recombinant outer membrane protein Oms28 of Borrelia burgdorferi. *J. Bacteriol.* **178**, 4909–4918 (1996).

7. Mulay, V. *et al.* Borrelia burgdorferi BBA74, a Periplasmic Protein Associated with the Outer Membrane, Lacks Porin-Like Properties. *J. Bacteriol.* **189**, 2063–2068 (2007).

8. Pal, U. *et al.* TROSPA, an Ixodes scapularis Receptor for Borrelia burgdorferi. *Cell* **119**, 457–468 (2004).

9. Figlerowicz, M., Urbanowicz, A., Lewandowski, D., Jodynis-Liebert, J. & Sadowski, C. Functional Insights into Recombinant TROSPA Protein from Ixodes ricinus. *PLoS ONE* **8**, e76848 (2013).

10. Tilly, K., Bestor, A. & Rosa, P. A. Functional Equivalence of OspA and OspB, but Not OspC, in Tick Colonization by Borrelia burgdorferi. *Infect. Immun.* **84**, 1565–1573 (2016).

11. Fingerle, V. *et al.* Dynamics of dissemination and outer surface protein Expression of different European Borrelia burgdorferi sensu lato strains in artificially infected Ixodes ricinus nymphs. *J. Clin. Microbiol.* **40**, 1456–1463 (2002).

12. Grimm, D. *et al.* Outer-surface protein C of the Lyme disease spirochete: A protein induced in ticks for infection of mammals. *Proc. Natl. Acad. Sci.* **101**, 3142–3147 (2004).

13. Norris, S. J., Carter, C. J., Howell, J. K. & Barbour, A. G. Low-passage-associated proteins of Borrelia burgdorferi B31: characterization and molecular cloning of OspD, a surface-exposed, plasmid-encoded lipoprotein. *Infect. Immun.* **60**, 4662–4672 (1992).

14. Bockenstedt, L. K., Wooten, R. M. & Baumgarth, N. Immune Response to Borrelia : Lessons from Lyme Disease Spirochetes. *Curr. Issues Mol. Biol.* 145–190 (2022) doi:10.21775/cimb.042.145.

15. Bernard, Q. *et al.* Plasticity in early immune evasion strategies of a bacterial pathogen. *Proc. Natl. Acad. Sci.* **115**, (2018).

16. Brissette, C. A. & Gaultney, R. A. That’s my story, and I’m sticking to it-an update on B. burgdorferi adhesins. *Front. Cell. Infect. Microbiol.* **4**, (2014).

17. Rössler, D. *et al.* Molecular and immunological characterization of the p83/100 protein of various Borrelia burgdorferi sensu lato strains. *Med. Microbiol. Immunol. (Berl.)* **184**, 23–32 (1995).

18. Margos, G. *et al.* MLST of housekeeping genes captures geographic population structure and suggests a European origin of Borrelia burgdorferi. *Proc. Natl. Acad. Sci. U. S. A.* **105**, 8730–8735 (2008).

19. Gürtler, V. & Stanisich, V. A. New approaches to typing and identification of bacteria using the 16S-23S rDNA spacer region. *Microbiol. Read. Engl.* **142 ( Pt 1)**, 3–16 (1996).
