# Supplementary figures and images for: Genetic landscape of Borrelia burgdorferi sensu stricto in Canada: a study of genetic diversity
Source: Sci Rep. 2025 Nov 20;15:40954. doi: 10.1038/s41598-025-24758-2 (PMC12635250; doi:10.1038/s41598-025-24758-2)

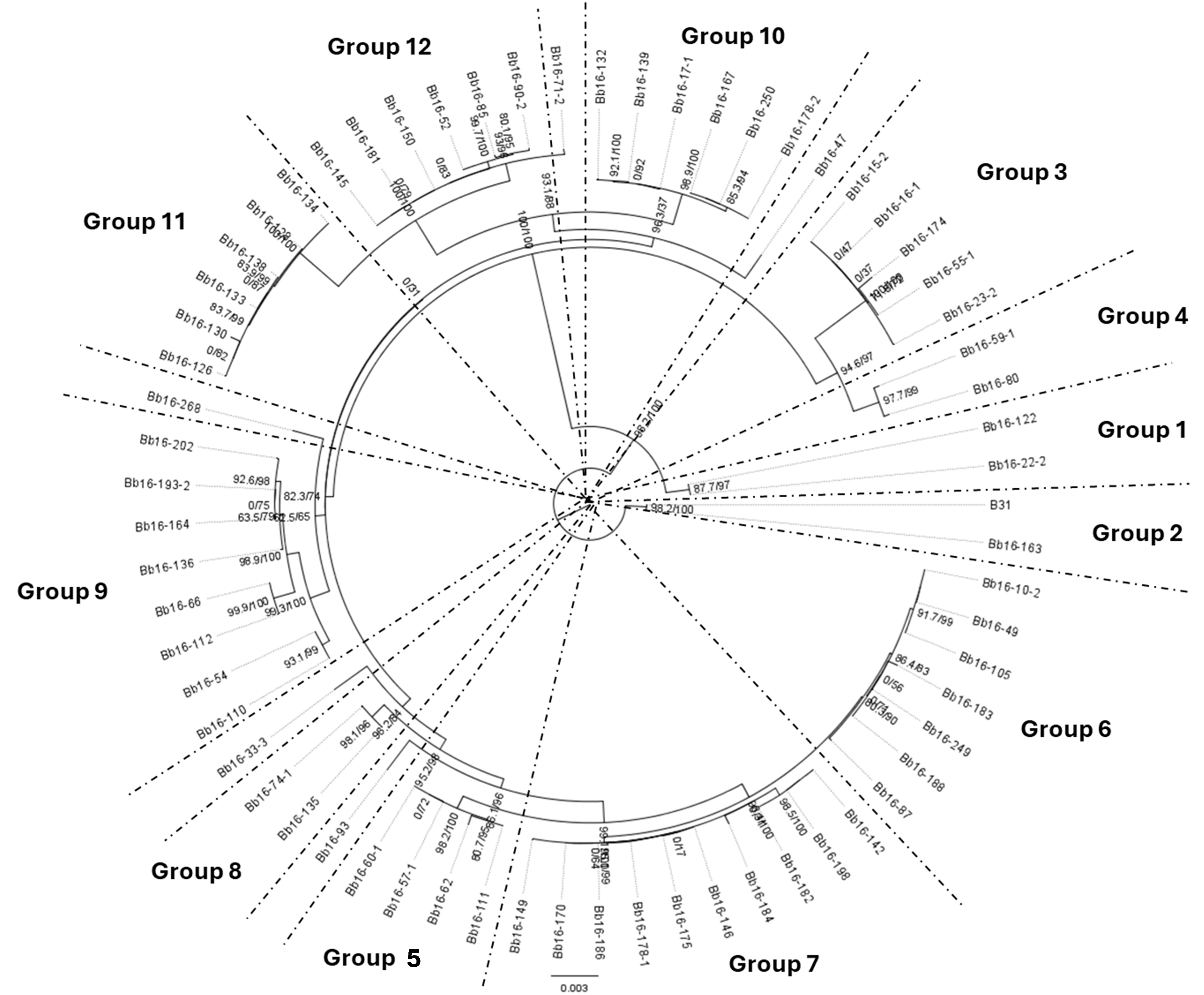

Supplement: Supplementary file 2 — Supplementary Material 2 [file 41598_2025_24758_MOESM2_ESM.tif]

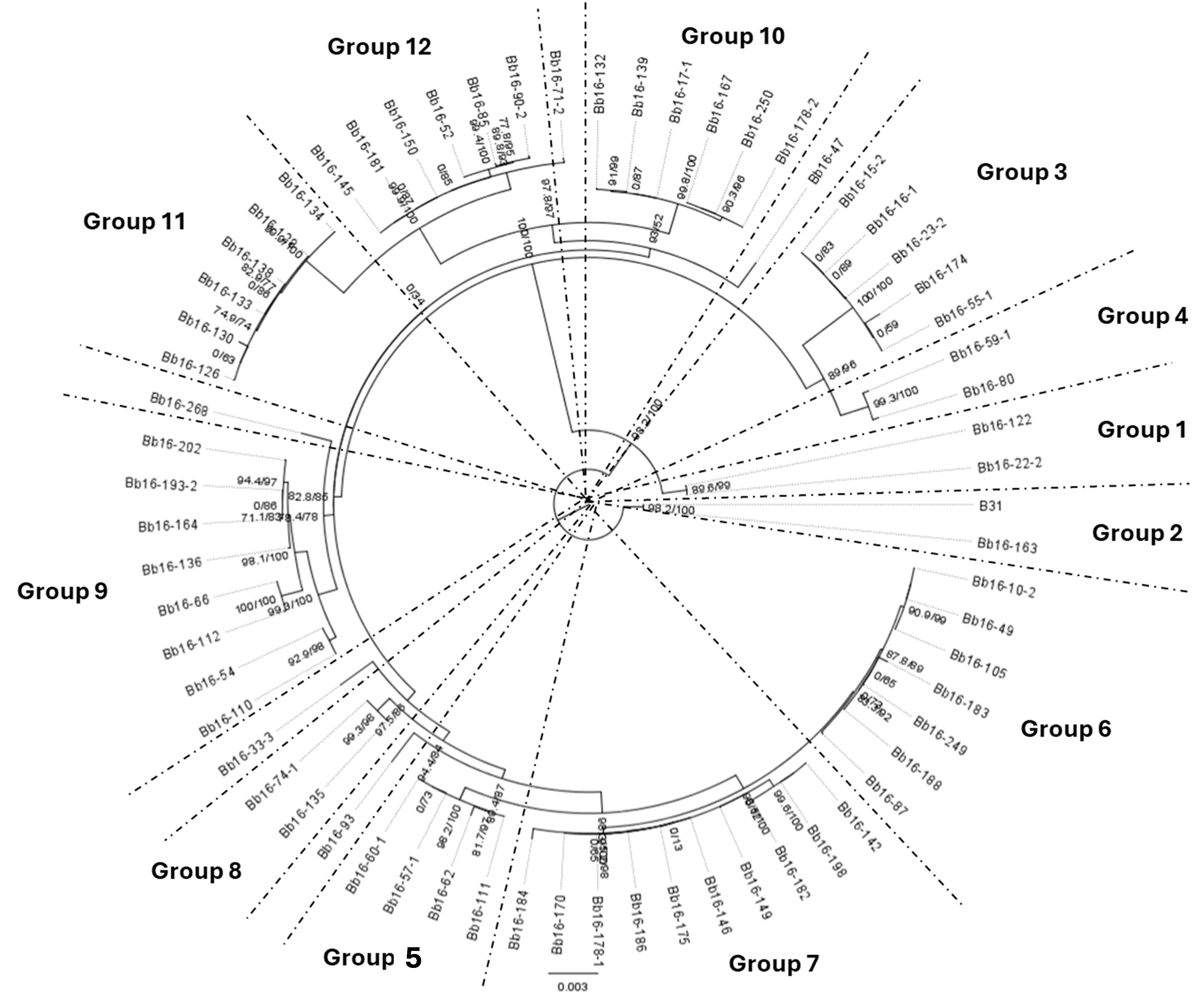

Supplement: Supplementary file 4 — Supplementary Material 4 [file 41598_2025_24758_MOESM4_ESM.tif]
